# The minor T allele of the MUC5B promoter rs35705950 associated with susceptibility to idiopathic pulmonary fibrosis: a meta-analysis

**DOI:** 10.1038/s41598-021-03533-z

**Published:** 2021-12-14

**Authors:** Xiaozheng Wu, Wen Li, Zhenliang Luo, Yunzhi Chen

**Affiliations:** Department of Preclinical Medicine, Guizhou University of Traditional Chinese Medicine, Guiyang, 510025 China

**Keywords:** Clinical genetics, Genotype, Medical genetics, Genetics, Respiratory tract diseases, Medical research, Genetics research

## Abstract

MUC5B promoter rs35705950 T/G gene polymorphism has been associated with the risk of IPF, but the influence of this relationship varies among different populations. In the past 2 years, there were new clinical studies with different results, but none of them reached unified conclusions. Therefore, this study further included the latest case–control studies, integrated their results and carried out meta-analysis on them to draw reliable conclusions. PubMed, EMBASE, CNKI, Wanfang database and VIP Chinese science were searched by a computer to collect the related literatures of MUC5B gene polymorphism and IPF susceptibility published before June 15, 2021. The first author, year of publication, diagnostic criteria and gene frequency were extracted after screened them. Forest plot was drawn and the trial sequential analysis (TSA) was carried out to confirm the stability of the meta-analysis results. Registration number: CRD42021272940. A total of 24 case–control studies (13 studies on the Caucasian, 7 studies on the Asian and 4 studies on the mixed population), and a total of 6749 IPF patients and 13,898 healthy controls were included in this study. The T vs.G, TT vs. GG, GT vs. GG, GT + TT vs. GG and TT vs. GG + GT genetic models of MUC5B promoter rs35705950 T/G polymorphism were associated with IPF risk in all populations, and the effect values were ([OR] 4.12, 95% CI [3.64, 4.67]), ([OR] 10.12, 95% CI [7.06, 14.49]), ([OR] 4.84, 95% CI [3.85, 6.08]), ([OR] 4.84, 95% CI [3.79, 6.19]) and ([OR] 5.11, 95% CI [4.02, 6.49]), respectively. The results of TSA confirmed the stability of the results. Subgroup analysis showed that T vs.G, TT vs. GG, GT vs. GG, GT + TT vs. GG and TT vs. GG + GT genetic models of MUC5B polymorphism were associated with IPF risk in Caucasian population. The effect values were ([OR] 4.50, 95% CI [3.93, 5.16]), ([OR] 10.98, 95% CI [7.59, 15.89]), ([OR] 6.27, 95% CI [5.37, 7.32]), ([OR] 6.30, 95% CI [5.19, 7.64]) and ([OR] 5.15, 95% CI [4.01, 6.61]), respectively. Similar results were also found in Asian and mixed populations. The association strength of the minor T allele in the Caucasian was more significant than that of the Asian population ([OR] 4.50 vs. [OR] 2.39), and the association strength of all genetic models carrying "T" was more significant than that of the Asian population ([OR] 10.98 vs. [OR] 4.29). In Caucasian, Asian and mixed populations, T minor allele carriers were more likely to be susceptible to pulmonary fibrosis, and TT genotype carriers were more likely to be susceptible to IPF than GT genotype carriers. The association between IPF and Caucasian population with minor T allele and all "T" genetic model was more significant than that of Asian population.

## Introduction

Idiopathic pulmonary fibrosis (IPF) was a disease with insidious onset, short course and high mortality^1,2^. In recent years, the incidence of IPF has been increasing, and the prevalence increased from 276 cases per 100,000 in 2010 to 725 cases per 100,000 in 2019^3^. At present, the cause of IPF is not yet clear, but the role of genetic factors has received more and more attention in the studies of its pathogenesis. A study had shown that as many as one fifth of patients affected by IPF reported that their family members had pulmonary fibrosis^4^. Mucin 5B (MUC5B) played an important role in immune regulation in maintaining bronchoalveolar epithelial function, and its genetic variation had been identified as a risk factor for IPF^5,6^.So far, Genome-wide Association Study (GWAS) has found a SNP in the promoter region of MUC5B gene (rs35705950), in which the T allele frequency was 30–35% in IPF cases^6–14^.Variant rs35705950 alone explains 5.9–9.4% of disease liability in the general population and 13.5% in people > 65 years of age^15^. MUC5B promoter SNP rs35705950 and its enhanced expression in distal airway epithelial cells are considered to be related to the pathogenesis of IPF^16–19^. Due to excessive lung injury and abnormal repair, excessive release of MUC5B can lead to the exacerbation of IPF^14,20–22^.SNP rs35705950 is a G to T trans version in the 5 'flanking region of MUC5B 3 kb upstream of the transcription initiation site, it shows the strongest association with IPF^14^.In the Caucasian population, 41.9% of IPF patients and 10.8% of the control group had the T-risk allele^11^, while in the Chinese population, the frequency of T allele was about 3.33% in IPF patients and 0.66% in the control group^23^. In recent years, meta-analysis has showed that comparing with G allele, minor T allele was associated with increased risk of IPF, TT genotype carriers were more prone to IPF than GT genotype carriers, and the association strength of Caucasian population was more significant than that of Asian population^24–26^.However, in the past two years, there were new clinical studies reporting the correlation between rs35705950 and IPF, and their research results were different, so there was no unified statement.

Therefore, based on the results of previous clinical studies, this study further included the latest case–control studies, integrated their results and carried out meta-analysis on them to draw reliable conclusions.

## Data and methods

This study has been registered in PROSPERO, registration number: CRD 42021272940 (https://www.crd.york.ac.uk/prospero/). The procedure of this protocol is based on PRISMA-P guidance^27^.

### Inclusion and exclusion criteria

#### Inclusion criteria

(1) The case–control studies are all based on the susceptibility of MUC5B rs35705950 T/G gene polymorphism and IPF; the language is either Chinese or English; the description of detection methods and means is accurate; (2) They conform to the authoritative standards established by the Chinese Society of Respiratory Medicine^2^ or the ATS/ERS/JRS/ALAT^1^.The patients were not limited in gender, age, race and nationality, and other serious systemic diseases were excluded; (3) The gene frequency data is complete and can be used to calculate the OR and 95% CI; (4) The distribution of genotype frequency of all subjects conformed to Hardy–Weinberg equilibrium^28^; (5) The score of Newcastle Ottawa scale (NOS)^29^ was no less than 7(≧7).

#### Exclusion criteria

Conference reports, reviews, case reports, the study failing to obtain allele frequency and research based on pedigree were not included. The same study published many times, only the one with the largest sample size and the most complete information was reserved.

### Outcomes

The pre-specified primary outcomes were to investigate whether MUC5B T/G increased the risk of IPF in the entire population. The secondary outcomes were to determine whether there was a difference in the strength of the association between MUC5B T/G and IPF among different ethnic groups.

### Retrieval strategy

PubMed, EMbase, CNKI, WanFang Database, VIP were searched through the Internet to collect domestic and foreign related literature data published before June 15, 2021on MUC5B gene polymorphism and IPF susceptibility. Theme words and keywords were retrieved combining with literature retrospective and manual retrieval methods. Search terms: "rs35705950" or"MUC5B"and"polymorphism"and"Idiopathic pulmonary fibrosis" or "IPF". The languages were limited to Chinese and English. Table S1 in supplemental content shows the search strategies in PubMed.

### Literature screening and data extraction

Two relatively independent researchers (X–Z W and W L) conducted literature screening and data extraction. After excluding the studies that obviously did not meet the inclusion criteria, they further read the full text of the studies that might meet the inclusion criteria to determine whether they could really be included, and then they cross checked them. They discussed to resolve or submitted to the third party (Y–Z C) for ruling when there were different opinions. If the report was not clear or lack of information, they tried to contact the author of the original text by e-mail to further obtain relevant data. The extracted data include: general clinical data, research subjects, year of publication, country of research subjects, race of research subjects, diagnostic criteria of IPF, number of cases in case group and healthy control group, and frequency of each genotype.

### Literature quality evaluation

Two independent researchers (X–Z W and W L) evaluated the selected literatures according to NOS^29^. Quality score ranged from 0 to 9, and the score of more than 7 were considered as high-quality ones.

### Statistical methods

Revman5.3 (Review Manager (RevMan)Version 5.3. Copenhagen: The Nordic Cochrane Centre, The Cochrane Collaboration, 2014. URL link: https://training.cochrane.org/ and stata12.0 (Stata/SE 12.0) for Windows (32-bit). Revision 22Apr 2015.Copyright 1985–2015 StataCorp LP. URL link: http://www.stata.com software were used to process data; Hardy–Weinberg genetic balance of subjects was analyzed by Pearson test; the heterogeneity between studies and subgroups was evaluated by Q-test and I^2^. If P < 0.1 or I^2^ > 50%, it was considered that there was heterogeneity between studies. The data were combined by random effect model, and OR and 95% CI were calculated; If there was no heterogeneity between studies and subgroups, the fixed effect model would be used for data consolidation; The OR value was calculated according to the data of allele genetic model (T vs. G), dominant genetic model (GT + TT vs. GG), recessive genetic model (TT vs. GG + GT), additive genetic model (TT vs. GG) and heterozygous genetic model (GT vs. GG);The forest plot was drawn to show the research results and their characteristics; Publication bias was judged by funnel plot, and evaluated by Begg's test and Egger's test. Sensitivity analysis was performed for the results with high heterogeneity. With reference to previous studies^30,31^, we used TSA software (Trial Sequential Analysis Viewer (TSA Viewer). Version 0.9.5.10 Beta. Copenhagen: Copenhagen Trial Unit, 2016. URL link: https://ctu.dk/tsa/ to perform TSA tests to evaluate the stability of the conclusion (Type I error) probability = 5%, statistical test power = 80%, relative risk reduction = 20%).

### Ethics and dissemination

This review does not require ethical approval because the included studies are published data and do not involve the patients’ privacy. The results of this review will be reported in accordance with the PRISMA extension statement and disseminated to a peer-reviewed journal.

## Results

### Literature search results

**Figure 1:**
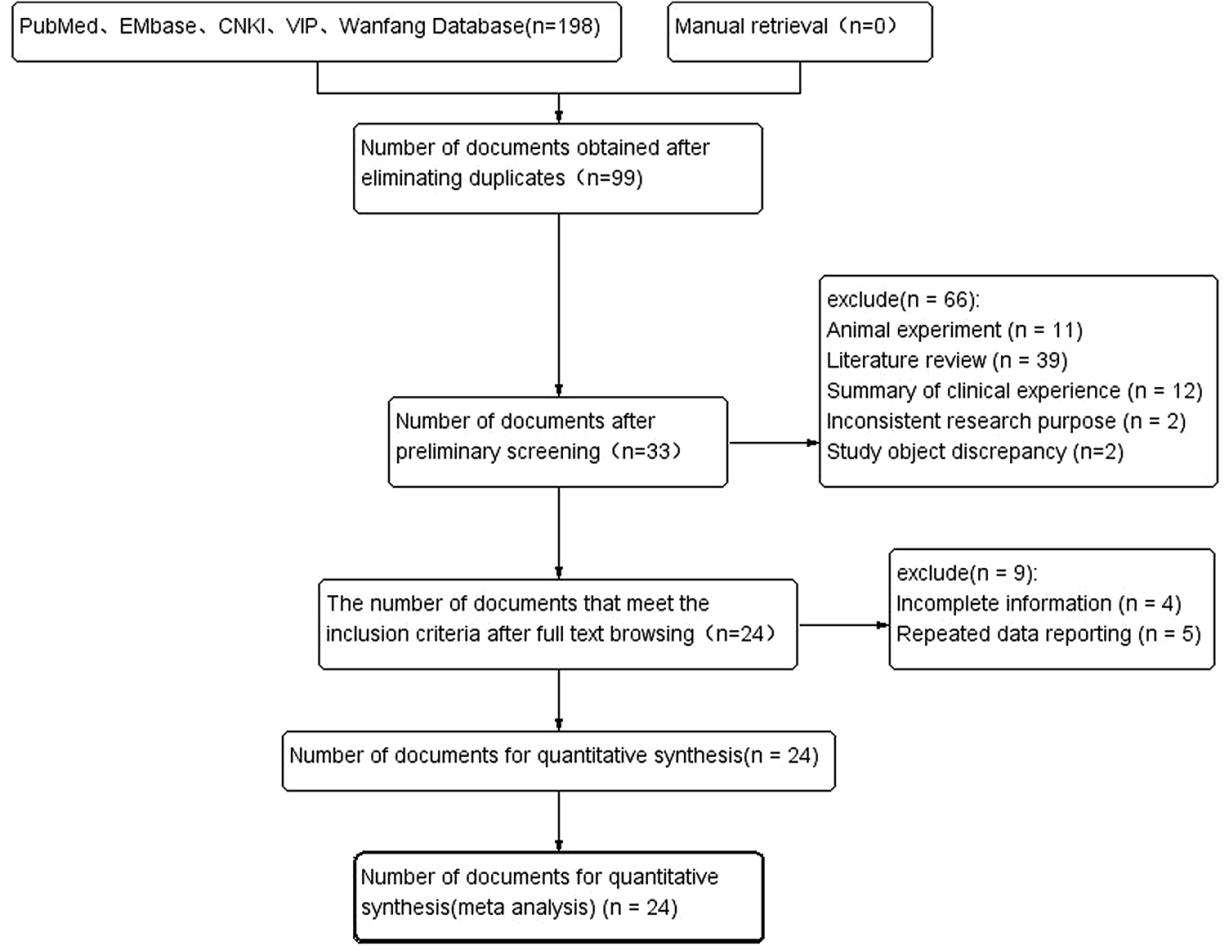
PRISMA literature screening flow chart.

The five databases were first detected with 198 related literatures, and after a layer by layer screening, 24 studies were finally included, including 13 studies in Caucasian, 7 in Asian and 4 in mixed population. A total of 6749 IPF patients and 13,898 healthy controls were included, including 5100 Caucasian, 1090 Asian and 559 mixed population patients. Fingerlin et al.^10^ was not included as a broad case definition was used (fibrotic IIP) and IPF specific results were not reported, meaning this paper did not meet our inclusion criteria. Figure 1 is about the flow chart of literature screening developed by PRISMA statement^27^, and Table 1 is about the basic features of the included studies.

**Table 1:** Basic features of the included study.

Studies	Year	Country	Ethnicity	Diagnostic criteria	Cases(N)		Gender (male/female)(N)		Age (years)		Genotyping method	HWE
					IPF	Control	IPF	Control	IPF	Control		
Allen^32^	2017	UK	Caucasian	2011,2015ATS/ERS/JRS/ALAT consensus statement	602	3366	426/176	2356 /1010	70 ± 8.4	65 ± 5.5	Affymetrix Axiom UK BiLEVE array	Yes
Bonella^33^	2021	Germany	Caucasian	2011,2018 ATS/ERS/JRS/ALAT consensus statement	62	50	43/8	37/13	63.5 ± 11	42 ± 2	TaqMan SNP Genotyping Assay	Yes
Borie^11^	2013	France	Caucasian	2001 ATS/ERS consensus statement	142	1383	116/26	–	69.86 ± 8.9	–	Taqman SNP genotyping assay-allelic discrimination method	Yes
Deng^34^	2018	China	Asian	2011 ATS/ERS/JRS/ALAT consensus statement	253	125	169/84	41/84	65.4 ± 11.1	65.3 ± 10.8	PCR	Yes
Dressen^35^	2018	USA	Caucasian	2011 ATS/ERS/JRS/ALAT consensus statement	1510	1874	1119/391	507/1367	67.29 ± 7.98	56.38 ± 9.32	Illumina X10 sequencers	Yes
Gou^36^	2020	China	Asian	2011 ATS/ERS/JRS/ALAT consensus statement	88	88	53/35	53/35	66.92 ± 5.80	66.30 ± 6.06	PCR	Yes
Helling^37^	2017	USA	Mixed	2013 ATS/ERS consensus statement	203	139	124/79	69/70	64 ± 8.3	57 ± 14.5	Taqman gene expression assay	Yes
HORIMASU (Asian)^13^	2014	Japanese	Asian	2002 ATS/ERS consensus statement	44	310	35/9	255/55	67.5 ± 1.6	50.6 ± 0.4	TaqMan SNP Genotyping	Yes
HORIMASU (Caucasian)^13^	2014	Germany	Caucasian	2002 ATS/ERS consensus statement	71	35	51/20	15/20	67.6 ± 1.2	44.3 ± 2.3	TaqMan SNP Genotyping	Yes
Jiang^38^	2015	China	Asian	2011 ATS/ERS/JRS/ALAT consensus statement	187	250	138/49	172/78	69.7 ± 4.3	67.7 ± 7.3	Taqman SNP genotyping	Yes
JOANNE^39^	2015	Netherland	Caucasian	2011ATS/ERS/JRS/ALAT 2001 ATS/ERS consensus statement	115	249	97/18	–	63.5 ± 11.0	–	TaqMan SNP genotyping assay	Yes
Kishore^40^	2016	Europe	Caucasian	2011 ATS/ERS/JRS/ALAT consensus statement	161	96	125/40	45/51	67.97 ± 11.60	34.45 ± 8.94	Sequenom Mass ARRAY	Yes
Ley (UCSF)^41^	2017	USA	Mixed	2011 ATS/ERS/JRS/ALAT consensus statement	147	503	–	–	–	–	Taqman SNP Genotyping assay	Yes
Ley (UTSW)^41^	2017	USA	Mixed	2011ATS/ERS/JRS/ALAT consensus statement	126	503	–	–	–	–	Taqman SNP Genotyping assay	Yes
Noth^6^	2013	USA	Caucasian	2000ATS/ERS consensus statement	1387	1367	1012/375	–	67 (61–73)	–	iPLEX Gold Platform	Yes
Peljto(Asian)^21^	2015	Korean	Asian	2011 ATS/ERS/JRS/ALAT consensus statement	239	87	60/179	–	65.1 ± 7.7	–	–	Yes
Peljto(Mexican)^21^	2015	Mexican	Mixed	2011 ATS/ERS/JRS/ALAT consensus statement	83	111	24/59	–	66.0 ± 7.7	–	–	Yes
Seibold^14^	2011	USA	Caucasian	ATS/ERS/JRS/ALAT consensus statement	492	322	352/140	147/175	67.2 ± 8.1	60.3 ± 12.6	Sequenom iPLEX assays	Yes
Song^42^	2021	China	Asian	2018ATS/ERS/JRS/ALAT consensus statement	114	100	79/35	59/41	65.13 ± 7.12	63.85 ± 6.67	PCR	Yes
Stock^9^	2013	UK	Caucasian	2000 2001ATS/ERS consensus statement	110	416	79/31	–	64.6 (45–85)	–	Taqman SNP Genotyping assay PCR	Yes
Stock^43^	2020	UK	Caucasian	2000 2001ATS/ERS consensus statement	23	20	–	–	–	–	Taqman SNP Genotyping assay	Yes
Wang^23^	2014	China	Asian	2011ATS/ERS/JRS/ALAT consensus statement	165	1013	101/64	525/488	61.78 ± 12.72	58.61 ± 12.72	Taqman SNP Genotyping assay	Yes
Wei^12^	2014	USA	Caucasian	2001 ATS/ERS consensus statement	84	689	55/29	360/329	64.4 ± 7.7	55.7 ± 13.2	Taqman SNP Genotyping assay PCR	Yes
Zhang^8^	2011	USA	Caucasian	2001 ATS/ERS consensus statement	341	802	238/103	436/366	67.9 ± 8.8	52.7 ± 14.7	Taqman SNP Genotyping assay	Yes

*ATS* American Thoracic Society, *ERS* European Respiratory Society, *JRS* Japanese Respiratory Society, *ALAT* Latin American Thoracic Society, *IPF* idiopathic pulmonary fibrosis, *PCR* polymerase chain reaction, *SNP* Single nucleotide polymorphism, *HWE* Harwin equilibrium.

Data are mean ± SD, or mean (IQR) or n, unless otherwise stated.

### Quality evaluation

24 trials in this study were evaluated by NOS scale. Except for the 2 trials that were moderate (scores = 7), all other trials had high results (scores = 8), indicating that the risk of bias was relatively low. The results are shown in Table S2 in supplemental content.

### Meta-analysis

#### T vs. G

**Figure 2:**
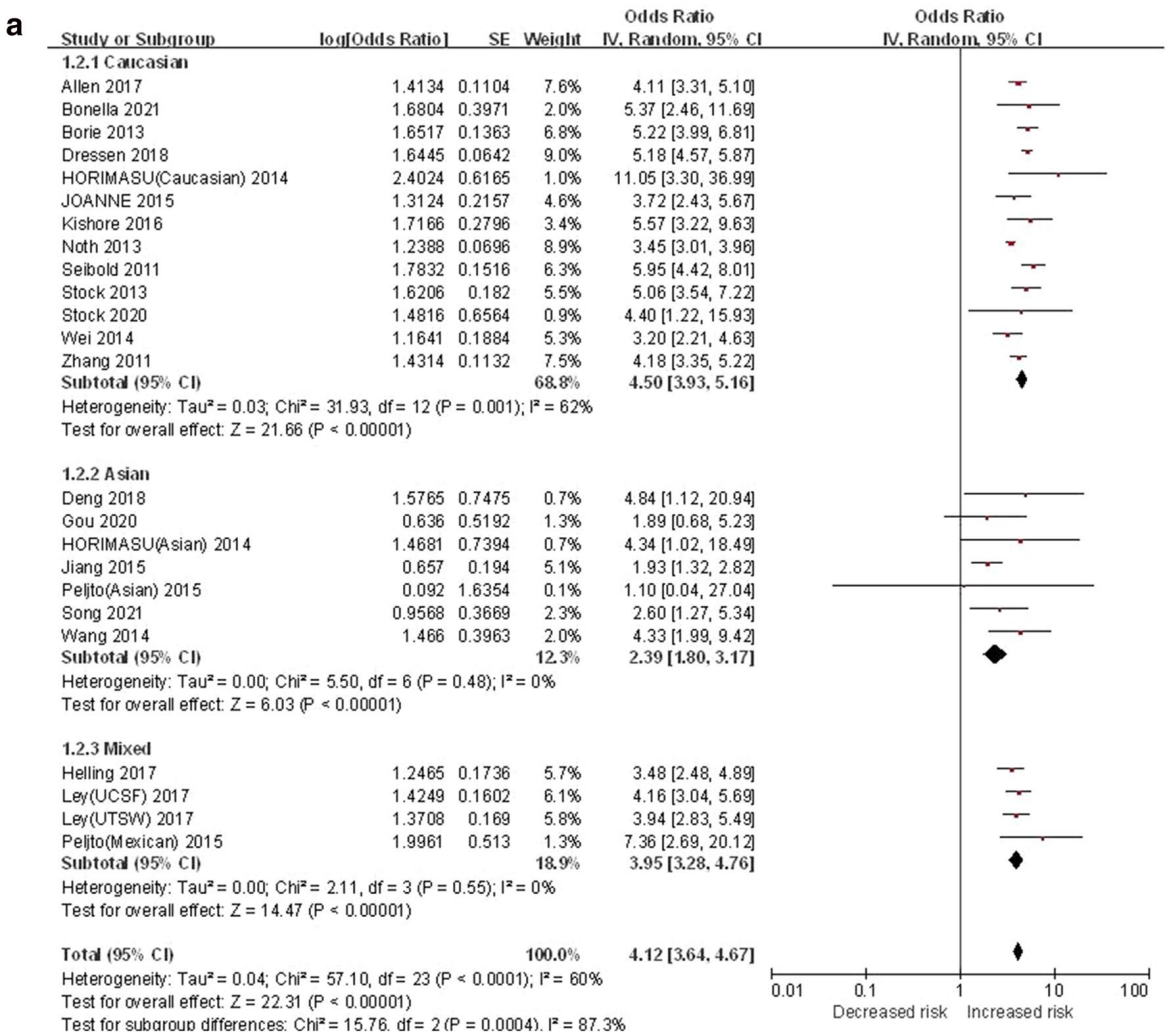

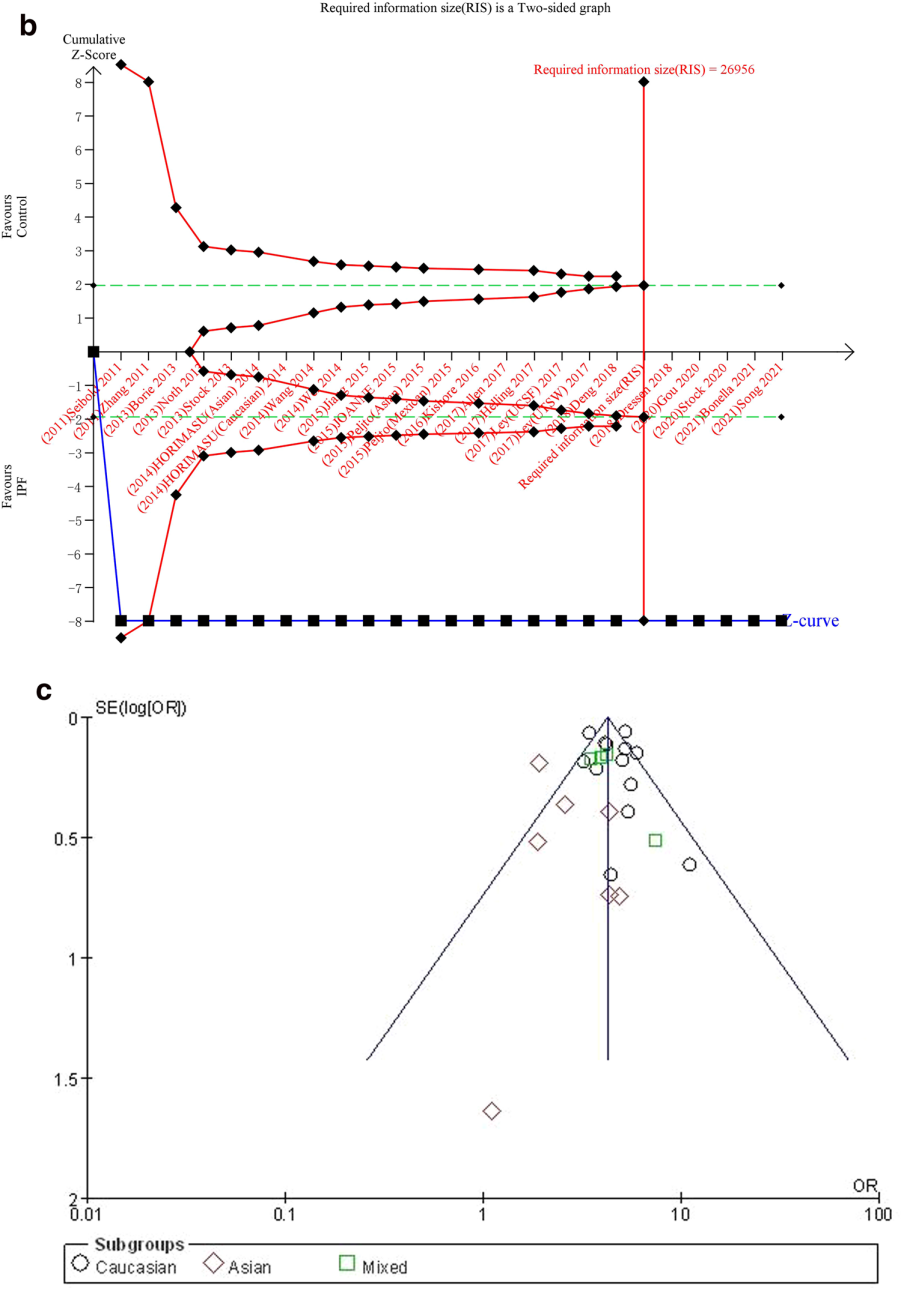
The T vs. G model was used to evaluate the correlation between MUC5B gene polymorphism and IPF susceptibility. (**a**) Forest plot of T vs. G genetic model. (**b**) Trial sequential analysis of MUC5B polymorphism and IPF risk using the allelic model (T vs. G) (Adjusted Boundaries Print). The combined sample size (N = 32,884) exceeded RIS (N = 26,956), the cumulative Z curve crossed the conventional boundary and the TSA boundary, and the association was established in advance. (**c**) Inverted funnel chart of T vs. G.

The allelic genetic model was used to evaluate the correlation between MUC5B gene polymorphism and IPF susceptibility. The heterogeneity test results showed that: P < 0.0001, I^2^ = 60%, and the T minor allele was associated with the risk of IPF compared with the G allele ([OR] 4.12, 95% CI [3.64, 4.67], P < 0.00001) (Fig. 2a). In the TSA, the calculated required information size (RIS) was 26,956. The combined sample size exceeded RIS, the cumulative Z curve crossed the conventional boundary and the TSA boundary, and the association was established in advance (Fig. 2b), indicating that further research will be not needed, because this significant correlation is unlikely to change. Sensitivity analysis results showed (Fig. S1a in supplemental content): The minimum value of all the research results was not lower than 1, indicating that there was no significant difference in the results after removing any one of the studies. The funnel chart was almost symmetrical, indicating that there was almost no obvious bias (Fig. 2c). The results of Begg's test (P = 0.785) and Egger's test (P = 0.683) suggested that there was no obvious bias as well (Fig. S1b,c in supplemental content).

After subgroup analysis of each population, the heterogeneity test results showed: Caucasian (P < 0.0001, I^2^ = 62%), Asian (P = 0.48, I^2^ = 0%), mixed (P = 0.55, I^2^ = 0%), each subgroup (P = 0.0004, I^2^ = 87.3%) (Fig. 2a).Compared with the G allele, the T allele was associated with the risk of IPF in various populations: Caucasian ([OR] 4.50, 95% CI [3.93, 5.16], P < 0.00001), Asian ([OR] 2.39, 95% CI [1.80, 3.17], P < 0.00001), mixed ([OR] 3.95, 95% CI [3.28, 4.76], P < 0.00001) (Fig. 2a). In the TSA, although the combined sample size of each subgroup did not exceed the RIS, the cumulative Z curve crossed the conventional boundary and the TSA boundary, which verified the stability of the meta-analysis results (Fig. S2a–c in supplemental content). Except for the fact that the funnel chart of the Caucasian was nearly symmetrical, the Asian and mixed populations were asymmetrical, indicating that there was a clear bias in the Asian and mixed populations (Fig. S2d–f in supplemental content). Although the results of the Begg's test and the Egger's test showed that there was no obvious bias among the various ethnic groups respectively (P > 0.05), overall there was a bias among the various ethnic groups (Egger's P = 0.035) (Table S3 in supplemental content).

#### TT vs. GG

**Figure 3:**
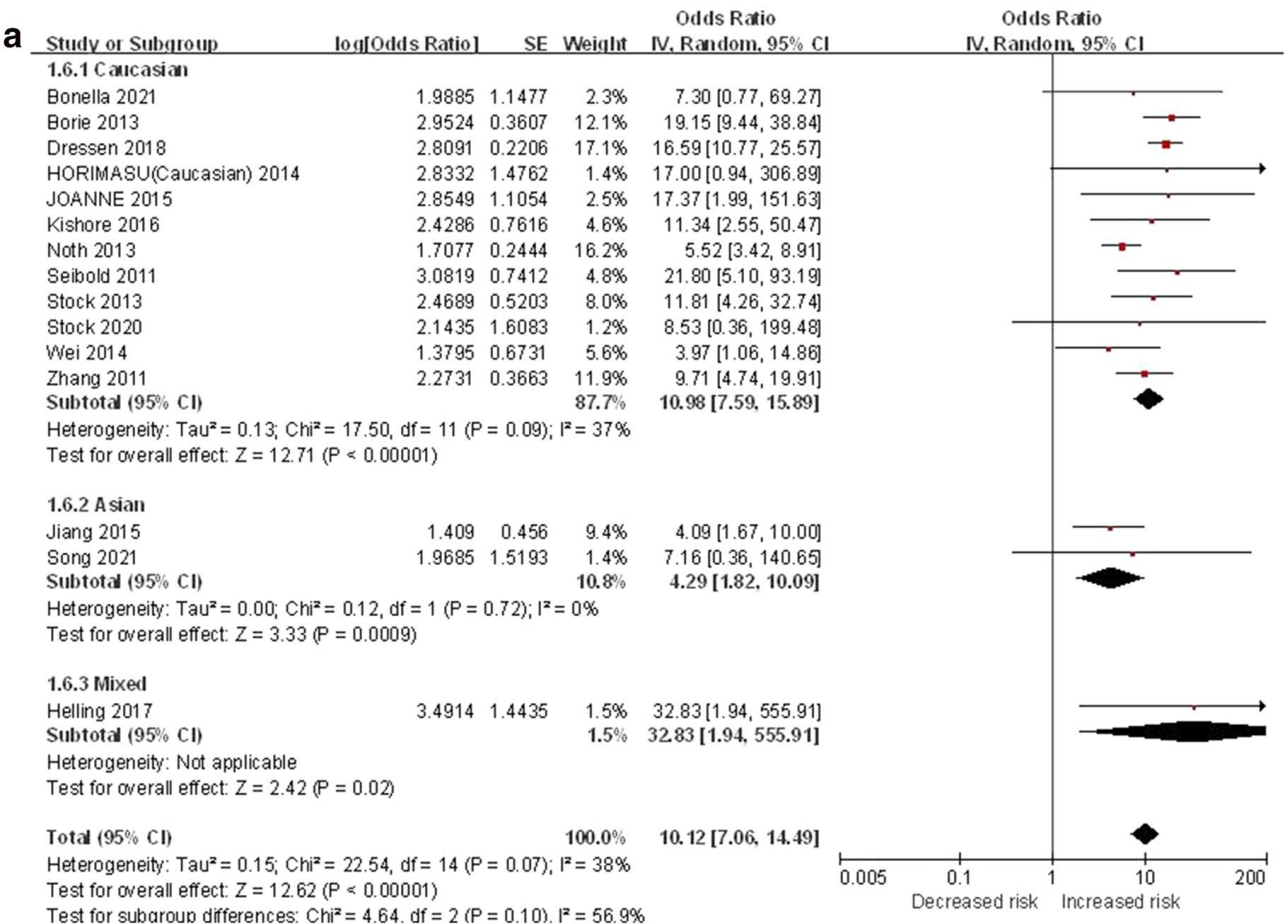

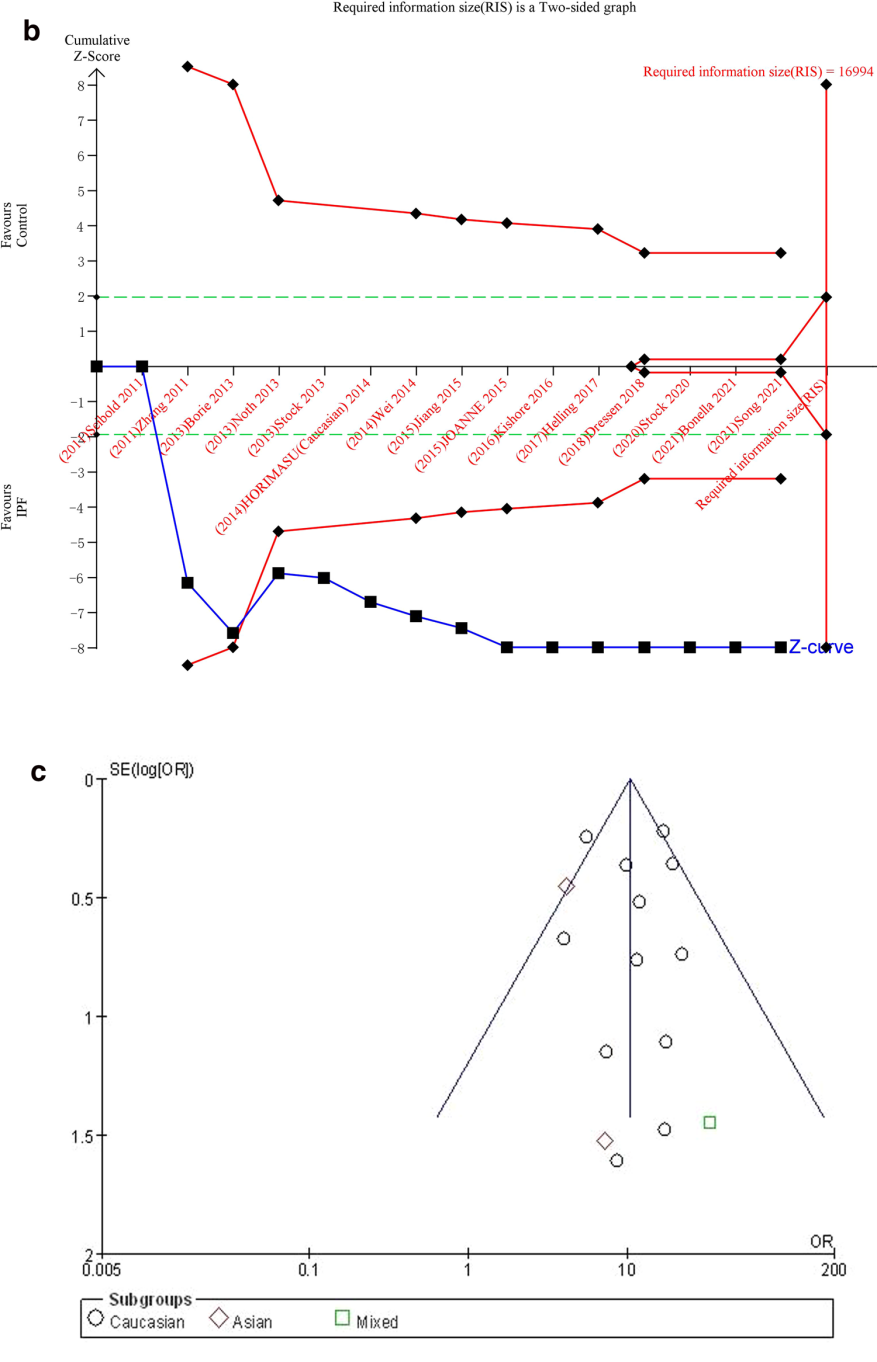
The TT vs. GG model was used to evaluate the correlation between MUC5B gene polymorphism and IPF susceptibility. (**a**) Forest plot of TT vs. GG genetic model. (**b**) Trial sequential analysis of MUC5B polymorphism and IPF risk using the additive genetic model (TT vs. GG) (Adjusted Boundaries Print). Although the combined sample size (N = 7690) did not exceed RIS (N = 16,994), the cumulative Z curve crossed the conventional boundary and the TSA boundary, and the association was established in advance. (**c**) Inverted funnel chart of TT vs. GG.

The additive genetic model was used to evaluate the correlation between MUC5B gene polymorphism and IPF susceptibility. The heterogeneity test results showed: P = 0.07, I^2^ = 38%, and the TT genotype was correlated with the risk of IPF compared with the GG genotype ([OR] 10.12, 95% CI [7.06, 14.49], P < 0.00001) (Fig. 3a).In the TSA, the calculated RIS was 16,994. Although the combined sample size did not exceed RIS, the cumulative Z curve crossed the conventional boundary and the TSA boundary, and the association was established in advance (Fig. 3b), indicating that further research will be not needed because this significant correlation is unlikely to change. Sensitivity analysis shows that all research results were stable and credible (Fig. S3a in supplemental content). The funnel chart was almost symmetrical, indicating that there was almost no obvious bias (Fig. 3c). The results of Begg's test (P = 0.921) and Egger's test (P = 0.965) suggested that there was no obvious bias (Fig. S3b,c in supplemental content).

After subgroup analysis of each population, the heterogeneity test results showed: Caucasian (P = 0.09, I^2^ = 37%), Asian (P = 0.72, I^2^ = 0%), each subgroup (P = 0.10, I^2^ = 56.9%), there was no heterogeneity test result (Fig. 3a) as there was only one literature reported in the mixed population. Compared with the GG genotype, the TT genotype was associated with the risk of IPF in all populations: Caucasian ([OR] 10.98, 95% CI [7.59, 15.89], P < 0.00001), Asian ([OR] 4.29, 95% CI [1.82, 10.09], P = 0.0009), mixed ([OR] 32.83, 95% CI [1.94, 555.91], P = 0.02) (Fig. 3a). In the TSA, the combined sample size of each subgroup did not exceed the RIS, but in the European population, the cumulative Z curve crossed the conventional boundary and the TSA boundary, which verified the stability of the meta-analysis results; The cumulative Z-curve in the Asian population only crosses the conventional boundary, and the cumulative Z-curve in the mixed population did not cross the conventional boundary and the TSA boundary, indicating that a larger sample size was needed to confirm the stability of the results (Fig. S4a–c in supplemental content).The funnel chart of the Caucasian was nearly symmetrical. Because the sample size of Asian and mixed populations was too small to judge their bias, this indicated that biases in Asian and mixed populations cannot be ruled out (Fig. S4d–f in supplemental content). Although the results of the Begg's test and the Egger's test showed that there was no obvious bias in Caucasian population (P > 0.05), the sample size of Asian and mixed populations was too small to judge their bias (Table S4 in supplemental content).

#### GT vs. GG

**Figure 4:**
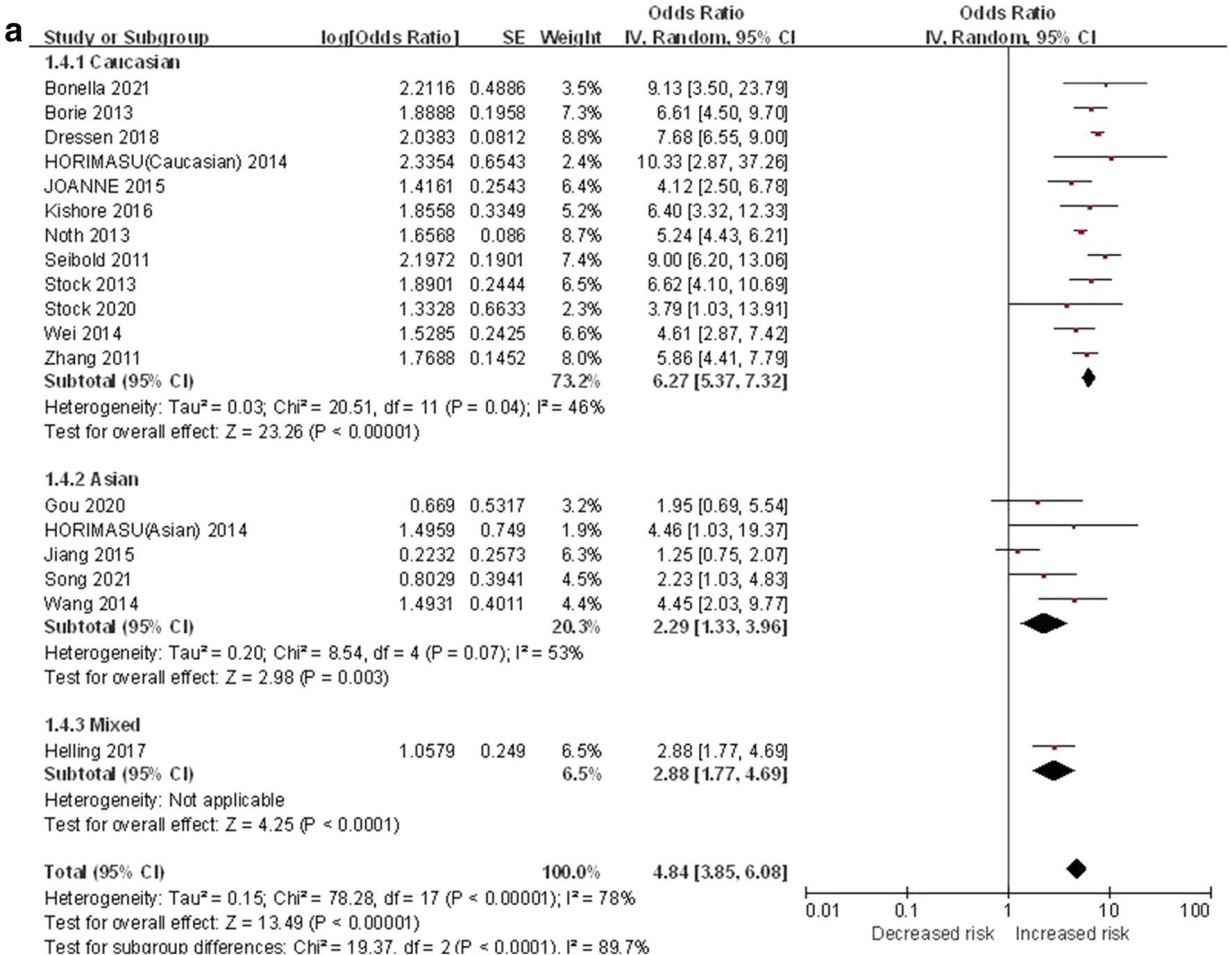

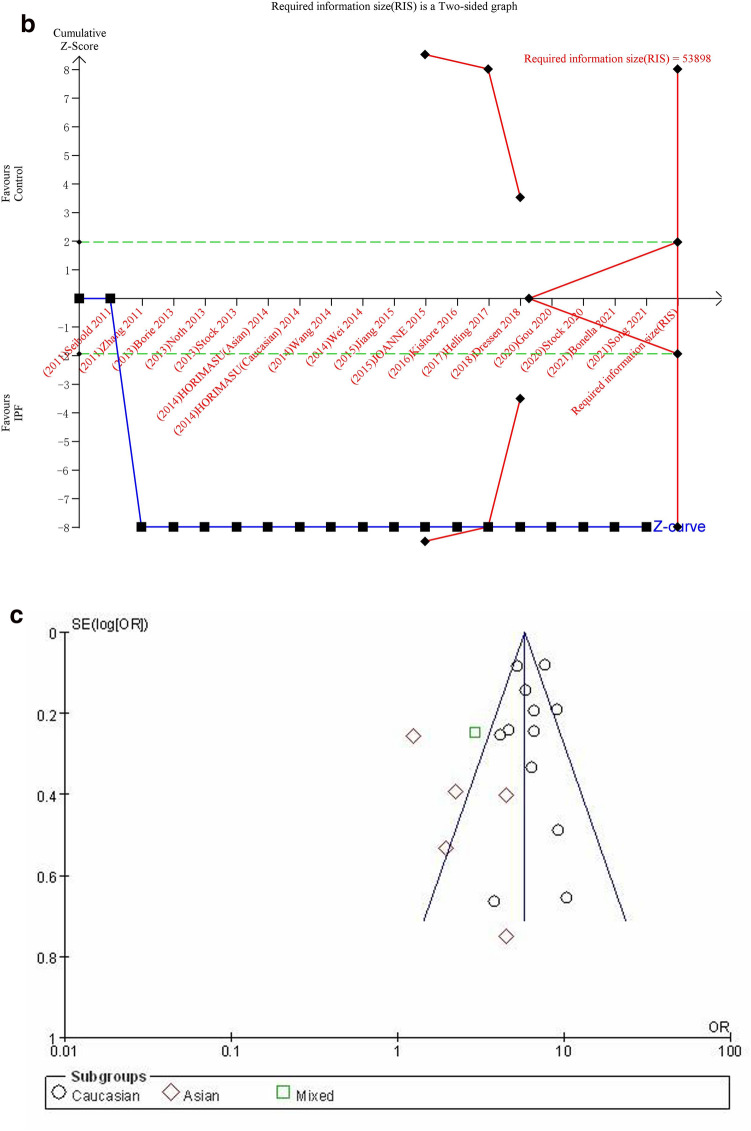
The GT vs. GG model was used to evaluate the correlation between MUC5B gene polymorphism and IPF susceptibility. (**a**) Forest plot of GT vs. GG genetic model. (**b**) Trial sequential analysis of MUC5B polymorphism and IPF risk using the heterozygous genetic model (GT vs. GG) (Adjusted Boundaries Print). Although the combined sample size (N = 12,737) did not exceed RIS (N = 53,898), the cumulative Z curve crossed the conventional boundary and the TSA boundary, and the association was established in advance. (**c**) Inverted funnel chart of GT vs. GG.

The heterozygous genetic model was used to evaluate the correlation between MUC5B gene polymorphism and IPF susceptibility. The heterogeneity test results showed that: P < 0.00001, I^2^ = 78%, and GT genotype was correlated with the risk of IPF compared with GG genotype ([OR] 4.84, 95% CI [3.85, 6.08], P < 0.00001) (Fig. 4a).In the TSA, the calculated RIS was 53,898, and the result was basically the same as TT vs. GG (Fig. 4b). Sensitivity analysis showed that all research results were stable and credible (Fig. S5a in supplemental content).The funnel chart was asymmetric (Fig. 4c), but Begg's test (P = 0.495) and Egger's test (P = 0.116) indicated that there was no obvious bias (Fig. S5b,c in supplemental content).

After subgroup analysis of each population, the heterogeneity test results showed: Caucasian (P = 0.04, I^2^ = 46%), Asian (P = 0.07, I^2^ = 53%), each subgroup (P < 0.0001, I^2^ = 89.7%), there was no heterogeneity test result (Fig. 4a) as there was only one literature reported in the mixed population. Compared with the GG genotype, the GT genotype was associated with the risk of IPF in all populations: Caucasian ([OR] 6.27, 95% CI [5.37, 7.32], P < 0.00001), Asian ([OR] 2.29, 95% CI [1.33, 3.96], P = 0.003), mixed ([OR]  2.88, 95% CI [1.77, 4.69], P < 0.0001) (Fig. 4a). In the TSA, the combined sample size of each subgroup did not exceed the RIS, but in the Caucasian population, the cumulative Z curve crossed the conventional boundary and the TSA boundary, which verified the stability of the meta-analysis results; In the Asian and mixed populations, the cumulative Z-curve only crossed the conventional boundary, indicating that a larger sample size would be needed to confirm the stability of the results (Fig. S6a–c in supplemental content).The funnel chart of the Caucasian was nearly symmetrical, and the Asian was asymmetric. Because the sample size of mixed population was too small to judge its bias, this indicated that bias in mixed population cannot be ruled out (Fig. S6d–f in supplemental content). Although the results of the Begg's test and the Egger's test showed that there were no obvious biases in Caucasian and Asian populations (P > 0.05), the sample size of mixed population was too small to judge its bias (Table S5 in supplemental content).

#### GT + TT vs. GG

**Figure 5:**
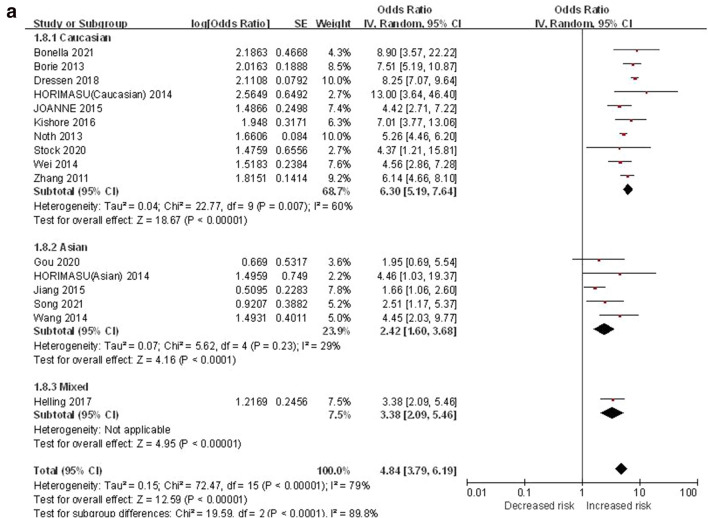

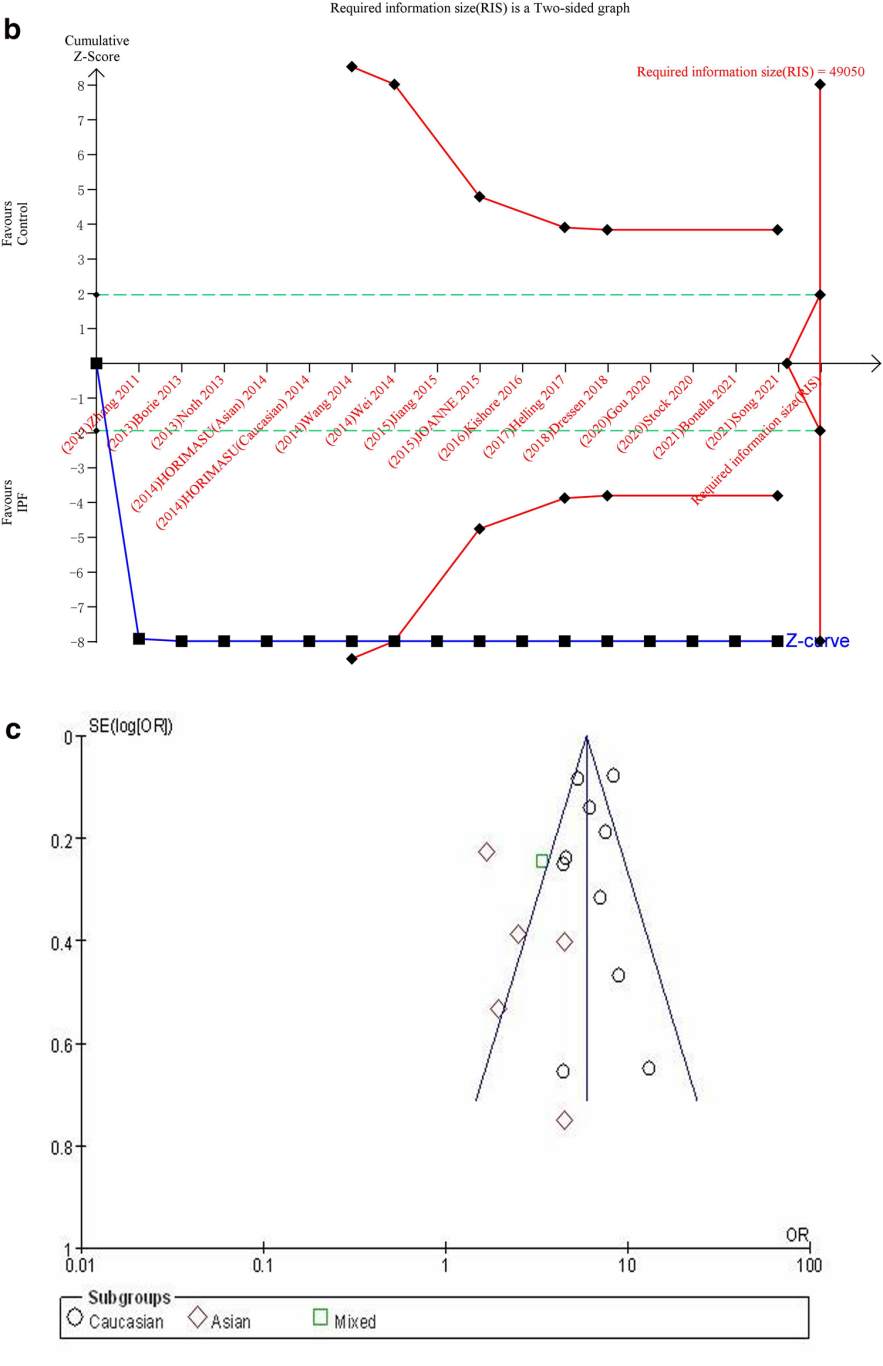
The GT + TT vs. GG model was used to evaluate the correlation between MUC5B gene polymorphism and IPF susceptibility. (**a**) Forest plot of GT + TT vs. GG genetic model. (**b**) Trial sequential analysis of MUC5B polymorphism and IPF risk using the dominant genetic model (GT + TT vs. GG) (Adjusted Boundaries Print). Although the combined sample size (N = 13,162) did not exceed RIS (N = 49,050), the cumulative Z curve crossed the conventional boundary and the TSA boundary, and the association was established in advance. (**c**) Inverted funnel chart of GT + TT vs. GG.

The dominant genetic model was used to evaluate the correlation between MUC5B gene polymorphism and IPF susceptibility. The heterogeneity test results showed: P < 0.00001, I^2^ = 79%, and the GT + TT genotype was correlated with the risk of IPF compared with the GG genotype ([OR] 4.84, 95% CI [3.79, 6.19], P < 0.00001) (Fig. 5a). In the TSA, the calculated RIS was 49,050, and the result was basically the same as TT vs. GG (Fig. 5b). Sensitivity analysis showed that all research results were stable and credible (Fig. S7a in supplemental content). The funnel chart was asymmetric (Fig. 5c), but Begg's test (P = 0.822) and Egger's test (P = 0.124) indicated that there was no obvious bias (Fig. S7b,c in supplemental content).

After subgroup analysis of each population, the heterogeneity test results showed: Caucasian (P = 0.007, I^2^ = 60%), Asian (P = 0.23, I^2^ = 29%), each subgroup (P < 0.0001, I^2^ = 89.8%), there was no heterogeneity test result (Fig. 5a) as there was only one literature reported in the mixed population. Compared with the GG genotype, the GT + TT genotype was associated with the risk of IPF in all populations: Caucasian ([OR] 6.30, 95% CI [5.19, 7.64], P < 0.00001), Asian ([OR] 2.42, 95% CI [1.60, 3.68], P < 0.0001), mixed ([OR] 3.38, 95% CI [2.09, 5.46], P < 0.00001) (Fig. 5a). TSA results were basically the same as GT vs. GG (Fig. S8a–c in supplemental content).The funnel chart of the Caucasian was nearly symmetrical, and the Asian was asymmetric. Because the sample size of mixed population was too small to judge its bias, this indicated that bias in mixed population cannot be ruled out (Fig. S8d–f in supplemental content). Although the results of the Begg's test and the Egger's test showed that there were no obvious biases in Caucasian and Asian populations (P > 0.05), the sample size of mixed population was too small to judge its bias (Table S6 in supplemental content).

#### TT vs. GG + GT

**Figure 6:**
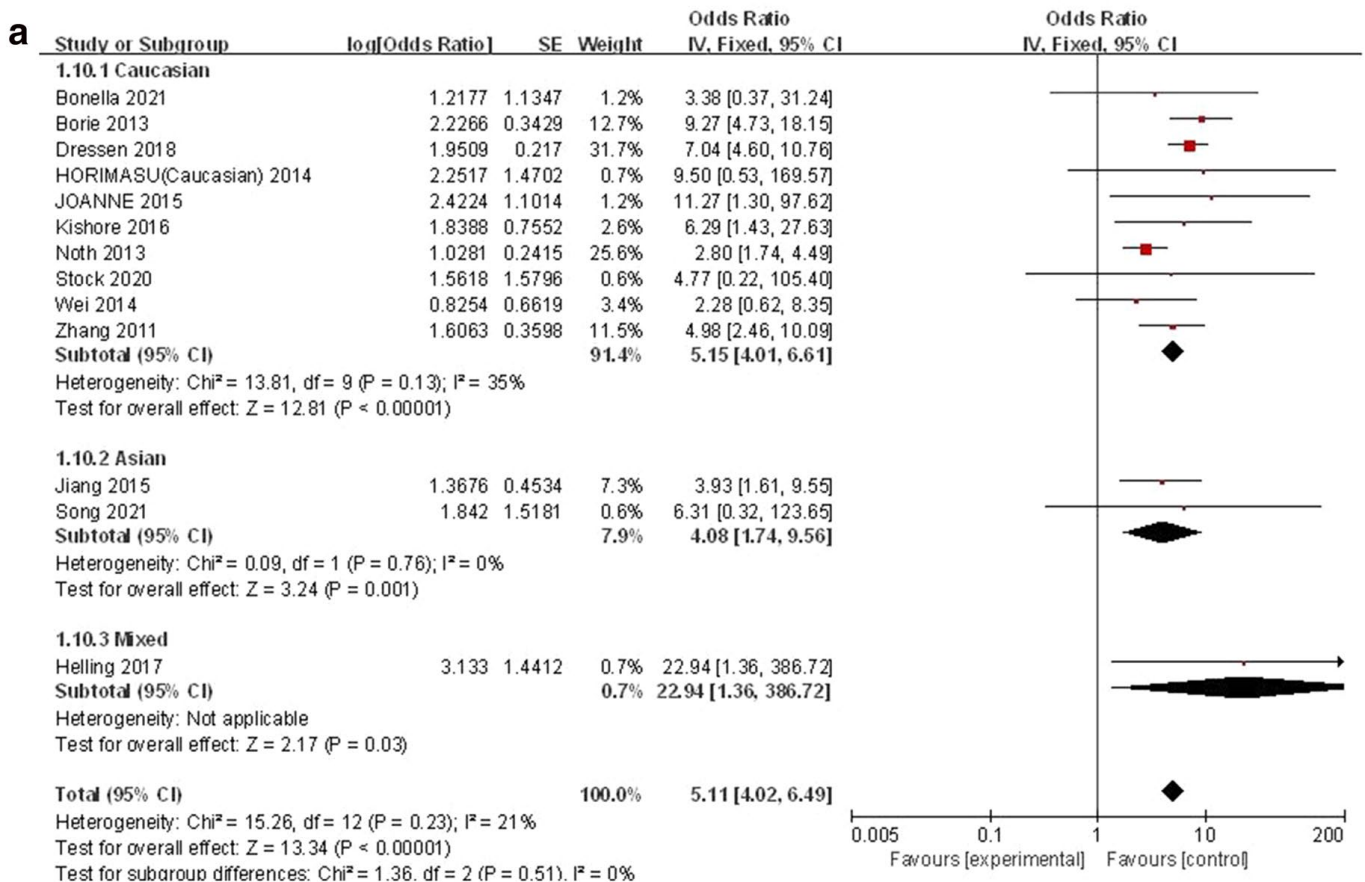

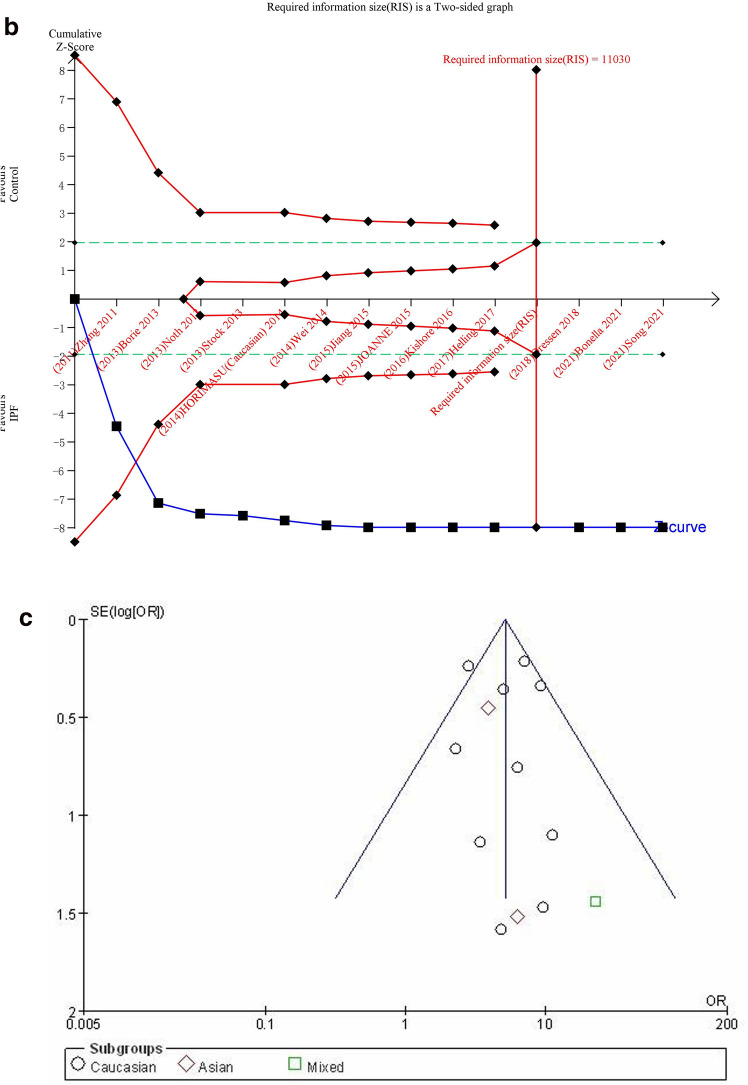
The TT vs. GG + GT model was used to evaluate the correlation between MUC5B gene polymorphism and IPF susceptibility. (**a**) Forest plot of TT vs. GG + GT genetic model. (**b**) Trial sequential analysis of MUC5B polymorphism and IPF risk using the recessive genetic model (TT vs. GG + GT) (Adjusted Boundaries Print). The combined sample size (N = 11,454) exceeded RIS (N = 11,030), the cumulative Z curve crossed the conventional boundary and the TSA boundary, and the association was established in advance. (**c**) Inverted funnel chart of TT vs. GG + GT.

The recessive genetic model was used to evaluate the correlation between MUC5B gene polymorphism and IPF susceptibility. The heterogeneity test results showed that: P = 0.23, I^2^ = 21%, and the TT genotype was correlated with the risk of IPF compared with the GG + GT genotype ([OR] 5.11, 95% CI [4.02, 6.49], P < 0.00001) (Fig. 6a). In the TSA, the calculated RIS was 11,030, and the result was basically the same as T vs. G (Fig. 6b). Sensitivity analysis showed that all research results were stable and credible (Fig. S9a in supplemental content). The funnel chart was almost symmetrical, indicating that there was almost no obvious bias (Fig. 6c). The results of Begg's Test (P = 0.951) and Egger's test (P = 0.679) also suggested that there was no obvious bias (Fig. S9b,c in supplemental content).

After subgroup analysis of each population, the heterogeneity test results showed: Caucasian (P = 0.13, I^2^ = 35%), Asian (P = 0.76, I^2^ = 0%), each subgroup (P = 0.51, I^2^ = 0%), there was no heterogeneity test result (Fig. 6a) as there was only one literature reported in the mixed population. Compared with the GG + GT genotype, the TT genotype was associated with the risk of IPF in all populations: Caucasian ([OR] 5.15, 95% CI [4.01, 6.61], P < 0.00001), Asian ([OR] 4.08, 95% CI [1.74, 9.56], P = 0.001), mixed ([OR] 22.94, 95% CI [1.36, 386.72], P = 0.03) (Fig. 6a). TSA results were basically the same as GT vs. GG (Fig. S10a–c in supplemental content).The funnel chart of the Caucasian was nearly symmetrical. Because the sample size of Asian and mixed populations was too small to judge their bias, this indicated that biases in Asian and mixed populations cannot be ruled out (Fig. S10d–f in supplemental content). Although the results of the Begg's test and the Egger's test showed that there was no obvious bias in Caucasian population (P > 0.05), the sample size of Asian and mixed populations was too small to judge their bias (Table S7 in supplemental content).

## Discussion

The incidence of Idiopathic pulmonary fibrosis (IPF) has been increasing year by year^3,44,45^. At present, the pathogenesis of IPF is mainly based upon repetitive injury to the alveolar epithelium and dysregulated repair^46–48^. So far, the data have identified that a variety of genetic mutations have been associated with IPF, such as TERT mutations^49,50^ and SFTPC and SFTPA mutations^51–53^. Among them, the MUC5B polymorphism (rs35705950) was also one of the most important genetic associations with IPF.GWAS found that the minor T allele of single nucleotide polymorphism (SNP) rs35705950 was present at a frequency of 38% in European subjects with IPF^14,37^. It has been found that the expression of MUC5B in subjects with IPF was 14.1 times higher than that in subjects without IPF^14^. Excessive production of MUC5B led to IPF due to excessive lung injury and abnormal repair^14,20,22,54^. Therefore, in recent 10 years, people have been studying the relationship between this gene polymorphism and IPF susceptibility. At present, some related studies have been published, but no consistent conclusion has been reached. Based on the meta-analysis, the data with the same research purpose can be summarized and analyzed, which increases the effectiveness of the test so as to draw more objective and reliable conclusions. Therefore, this study conducted a meta-analysis on the relationship between the polymorphism of MUC5B rs35705950 gene and the susceptibility to IPF.

A total of 24 case–control studies with 6749 IPF patients and 13,898 healthy controls were included. The genotype and allele distribution frequencies of all subjects were in accordance with Hardy Weinberg equilibrium test, which showed that the selection of subjects was representative of the population, and the samples were in a random distribution equilibrium and a wide range of population. The results showed that rs35705950 T/G polymorphism of MUC5B promoter was associated with IPF risk in T vs.G, TT vs. GG, GT vs. GG, GT + TT vs. GG and TT vs. GG + GT genetic models, meanwhile, sensitivity analysis and publication bias analysis showed that the results were stable and reliable. The results of test sequential analysis (TSA) also confirmed the stability of the results. These showed that people carrying the T minor allele were more likely to be susceptible to pulmonary fibrosis, and the T minor allele was a risk factor for the onset of pulmonary fibrosis. It was found that carriers of TT genotype ([OR] 10.12) were more likely to develop IPF than carriers of GT genotype ([OR] 4.84) when comparing the OR values of different genetic models.

Considering ethnic factors may have an impact on the results, we conducted a subgroup analysis: In the Caucasian population, the MUC5B gene polymorphism was in T vs. G, TT vs. GG, GT vs. GG, GT + TT vs. GG and TT vs. GG + GT genetic models were related to the risk of IPF, and the results of TSA and publication bias analysis confirmed the stability of the results. These showed that Caucasian populations carrying T minor alleles were more susceptible to pulmonary fibrosis, and T minor alleles were a risk factor for the onset of pulmonary fibrosis in the Caucasian population. TT genotype carriers ([OR] 10.98) were more likely to be susceptible to pulmonary fibrosis than GT genotype carriers ([OR] 6.27). Similar results were also found in Asian and mixed populations. It showed that Asian populations and mixed populations carrying T minor alleles were also susceptible to pulmonary fibrosis. T minor alleles were also the risk factors for the onset of pulmonary fibrosis in Asian populations and mixed populations. TT genotype carriers were more susceptible to IPF than GT genotype carriers (Asian: [OR] 4.29 vs. [OR] 2.29; mixed: [OR] 32.83 vs. [OR] 2.88). For the Caucasian population and the Asian population, the association strength of the minor T allele in the Caucasian was more significant than that of the Asian population ([OR] 4.50 vs. [OR] 2.39), and the association strength of all genetic models carrying "T" was more significant than that of the Asian population ([OR] 10.98 vs. [OR] 4.29).

The above conclusions were very similar to the results of clinical studies in the last year^33^: A recent retrospective study reported the susceptibility of 62 Caucasian IPF patients who were followed up from 2012 to 2019. The results showed that the MUC5B rs35705950 minor T allele was more common in IPF subjects than in healthy subjects (35% vs 9%, P < 0.001). In addition, Stock et al.^43^ observed increased expression of MUC5B in T allele carriers of Caucasian IPF patients (n = 23). A recent case–control study^42^ in Chinese Han population showed that the frequency of GT + TT genotype and T allele in patients was significantly higher than that in controls. The OR of IPF in T allele carriers was 2.603, 95% CI was (1.268–5.343). The average overall survival (OS) of patients with GT and TT genotypes of MUC5B rs35705950 was significantly lower than that of patients with GG. It is speculated that the polymorphism of MUC5B rs35705950 gene may be a risk factor for IPF in Chinese Han population, and the polymorphism of MUC5B rs35705950 gene was related to the decreased mortality of IPF patients.

Heterogeneity was generally considered to be the main factor affecting the reliability of meta-analysis results. In this study, it’s confirmed that there was heterogeneity except TT vs. GG + GT genetic model, and there was also some heterogeneity between subgroups. The heterogeneity of Caucasian population was obvious, but after sensitivity analysis, it was found that the results of each genetic model were relatively stable. Further publication bias analysis did not find any bias, indicating that the results of this study were generally stable and reliable. In terms of sample size, although the sample size of each genetic model in all populations basically met or approached the sample requirements of TSA. However, after subgroup analysis, the sample size of each population still did not meet the requirements of TSA, especially in Asian and mixed groups, which will affect the credibility of the results of Asian population and mixed population to a certain extent. Although the TSA results confirmed the stability of the T vs. G results of Asian and mixed groups, there was a certain publication bias in these results. The reason for this publication bias may be the relatively small sample size. In addition, for other genotypes in Asian populations and mixed populations, the relatively small sample size made it impossible to judge the stability and publication bias of these results. Therefore, for the results of Asian populations and mixed populations, more samples will be needed to confirm it. In addition, because the original studies included did not explore the content of gene–gene and gene–environment, and couldn’t collect relevant data, the interaction between environment and gene could not be further analyzed, which all caused the limitations of this meta-analysis.

## Conclusion

The meta-analysis overcame the difficulties mentioned above and reflected the relationship between IPF and different ethnic groups as much as possible. The meta-analysis results showed that people with T minor allele in Caucasian, Asian and mixed populations were more likely to be susceptible to pulmonary fibrosis, and those with TT genotype were more likely to be susceptible to IPF than those with GT genotype, moreover, the association strength of minor T allele in Caucasian population was more significant than that in Asian population, and the association strength of all genetic models carrying "T" was more significant than that in Asian population. But of course, more and higher quality large sample case–control studies will be needed to verify the results in Asian population to provide more effective basis for the prevention and treatment of IPF.

## Supplementary Information

Supplementary Information.

## Data Availability

Data supporting our findings are contained within the manuscript.

## Abbreviations

IPF: Idiopathic pulmonary fibrosis
MUC5B: Mucin 5B
CNKI: China national knowledge infrastructure
VIP: VIP Chinese science and technology periodical database
TSA: Trial sequential analysis
GWAS: Genome-wide association study
SNP: Single nucleotide polymorphism
OR: Odds ratio
95% CI: 95% Confidence interval
ATS: American thoracic society
ERS: European respiratory society
JRS: Japanese respiratory society
ALAT: Latin American thoracic society
NOS: Newcastle Ottawa scale
RIS: Required information size
OS: Average overall survival
PCR: Polymerase chain reaction
HWE: Hardy Weinberg equilibrium
