# Supplementary material for: The minor T allele of the MUC5B promoter rs35705950 associated with susceptibility to idiopathic pulmonary fibrosis: a meta-analysis
Source: Sci Rep. 2021 Dec 14;11:24007. doi: 10.1038/s41598-021-03533-z (PMC8671516; doi:10.1038/s41598-021-03533-z)
Supplement: Supplementary file 1 — Supplementary Information. [file 41598_2021_3533_MOESM1_ESM.docx]

Figure S1a Influence analysis results of T vs.G

Figure S1b T vs.G funnel chart generated by Begg's Test( Pr > |z| = 0.785 (continuity corrected))

Figure S1c T vs.G funnel chart of bias generation detected by Egger's test(P>|t|=0.683)

**Figure S1 Influence analysis, Begg's Test and Egger's test results of T vs.G**

**
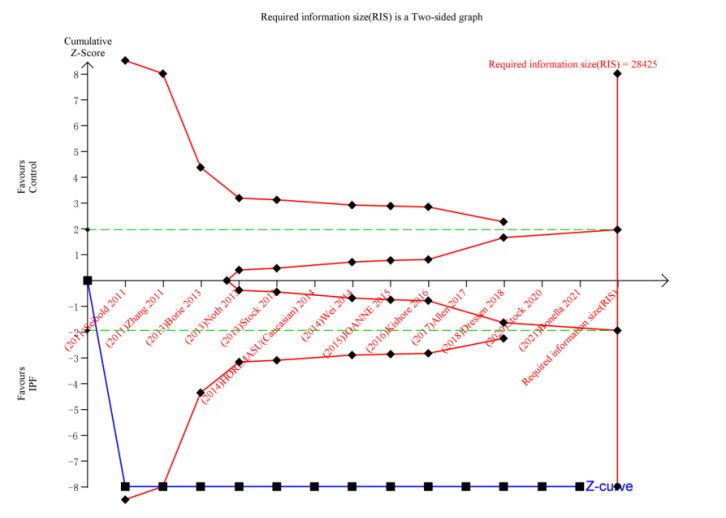
**

Figure S2a Trial sequential analysis of MUC5B polymorphism and IPF risk in Caucasian using the allelic model (T vs.G)

**
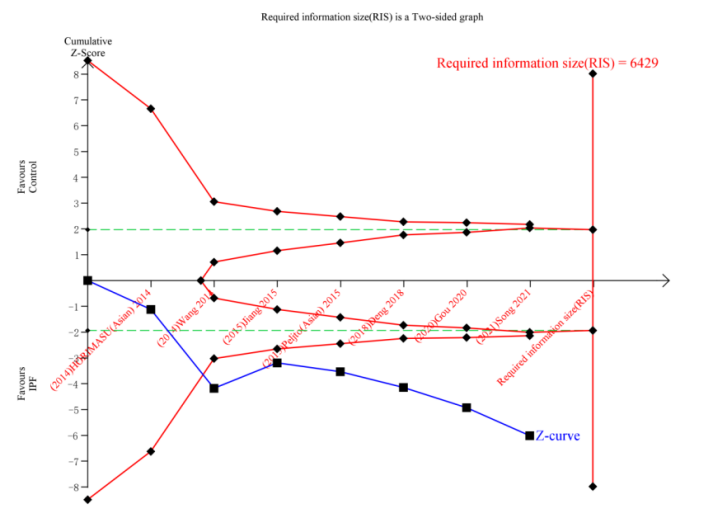
**

Figure S2b Trial sequential analysis of MUC5B polymorphism and IPF risk in Asian using the allelic model (T vs.G)

**
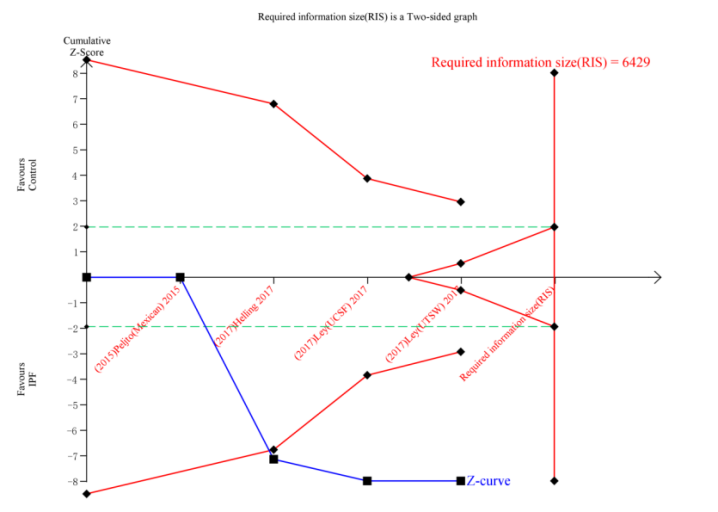
**

Figure S2c Trial sequential analysis of MUC5B polymorphism and IPF risk in Mixed using the allelic model (T vs.G)

Figure S2d Inverted funnel chart of T vs.G of Caucasian

Figure S2e Inverted funnel chart of T vs.G of Asian

Figure S2f Inverted funnel chart of T vs.G of Mixed

**Figure S2 MUC5B polymorphism and IPF risk in different populations using the allelic model (T vs.G)**

Note:Figure S2a-c:The Blue line was cumulative Z curve;The Red line was the Trial sequential monitoring(TSA) boundary or Required information size(RIS);The Green line was the Conventional boundary.(Adjusted Boundaries Print)

Figure S3a Influence analysis results of TT vs.GG

Figure S3b TT vs.GG funnel chart generated by Begg's Test(Pr > |z|=0.921 (continuity corrected))

Figure S3c TT vs.GG funnel chart of bias generation detected by Egger's test(P>|t|=0.965)

**Figure S3 Influence analysis, Begg's Test and Egger's test results of TT vs.GG**

**
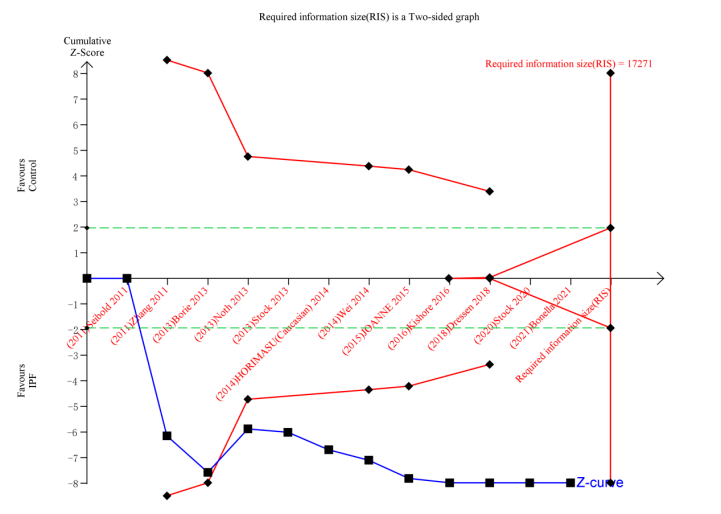
**

Figure S4a Trial sequential analysis of MUC5B polymorphism and IPF risk in Caucasian using the additive genetic model(TT vs.GG)

**
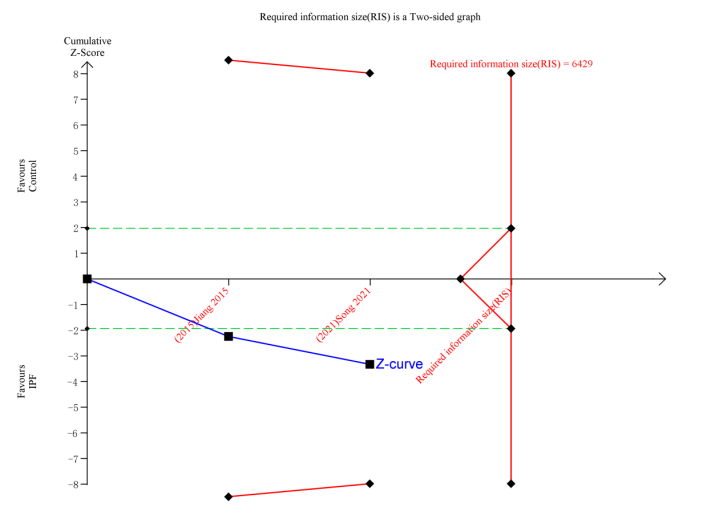
**

Figure S4b Trial sequential analysis of MUC5B polymorphism and IPF risk in Asian using the additive genetic model(TT vs.GG)

**
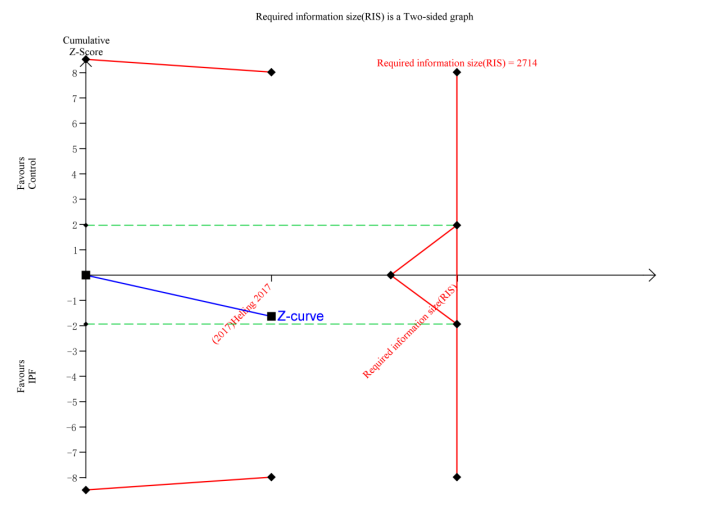
**

Figure S4c Trial sequential analysis of MUC5B polymorphism and IPF risk in Mixed using the additive genetic model(TT vs.GG)

Figure S4d Inverted funnel chart of TT vs.GG of Caucasian

Figure S4e Inverted funnel chart of TT vs.GG of Asian

Figure S4f Inverted funnel chart of TT vs.GG of Mixed

**Figure S4 MUC5B polymorphism and IPF risk in different populations using the additive model (TT vs.GG)**

Note:Figure S4a-c:The Blue line was cumulative Z curve;The Red line was the Trial sequential monitoring(TSA) boundary or Required information size(RIS);The Green line was the Conventional boundary.(Adjusted Boundaries Print)

Figure S5a Influence analysis results of GT vs.GG

Figure S5b GT vs.GG funnel chart generated by Begg's Test(Pr > |z|=0.495 (continuity corrected))

Figure S5c GT vs.GG funnel chart of bias generation detected by Egger's test(P>|t|=0.116)

**Figure S5 Influence analysis, Begg's Test and Egger's test results of GT vs.GG**

**
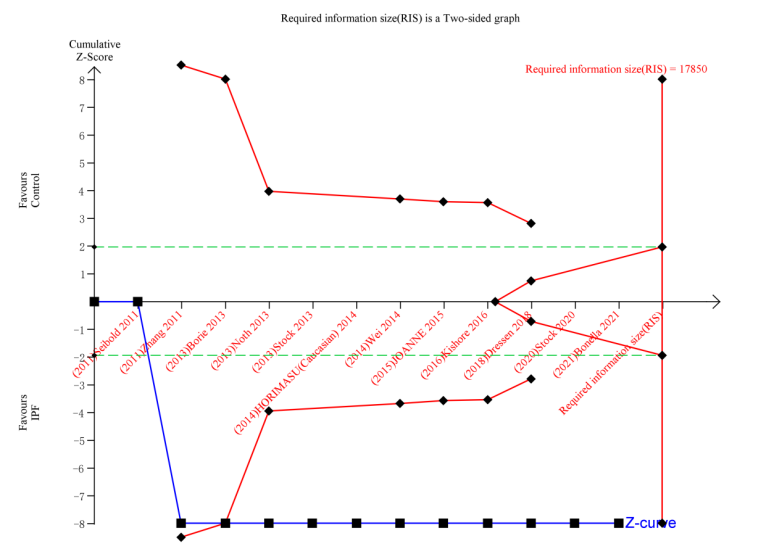
**

Figure S6a Trial sequential analysis of MUC5B polymorphism and IPF risk in Caucasian using the heterozygous genetic model(GT vs.GG)

**
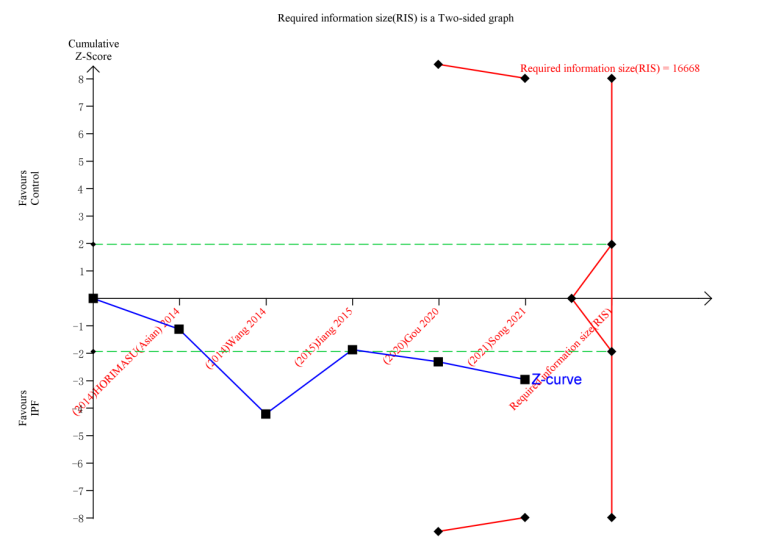
**

Figure S6b Trial sequential analysis of MUC5B polymorphism and IPF risk in Asian using the heterozygous genetic model(GT vs.GG)

**
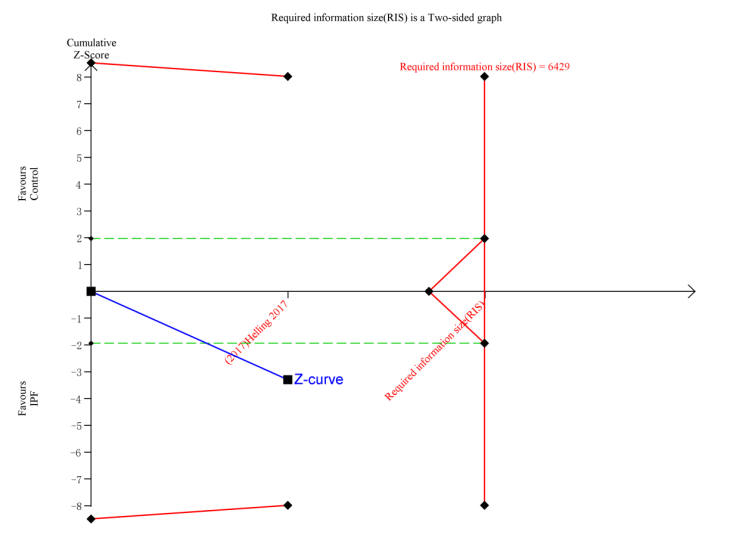
**

Figure S6c Trial sequential analysis of MUC5B polymorphism and IPF risk in Mixed using the heterozygous genetic model(GT vs.GG)

Figure S6d Inverted funnel chart of GT vs.GG of Caucasian

Figure S6e Inverted funnel chart of GT vs.GG of Asian

Figure S6f Inverted funnel chart of GT vs.GG of Mixed

**Figure S6 MUC5B polymorphism and IPF risk in different populations using the heterozygous model (GT vs.GG)**

Note:Figure S6a-c:The Blue line was cumulative Z curve;The Red line was the Trial sequential monitoring(TSA) boundary or Required information size(RIS);The Green line was the Conventional boundary.(Adjusted Boundaries Print)

Figure S7a Influence analysis results of GT+TT vs. GG

Figure S7b GT+TT vs. GG funnel chart generated by Begg's Test(Pr > |z|=0.822 (continuity corrected))

Figure S7c GT+TT vs. GG funnel chart of bias generation detected by Egger's test(P>|t|=0.124)

**Figure S7 Influence analysis, Begg's Test and Egger's test results of GT+TT vs.GG**

**
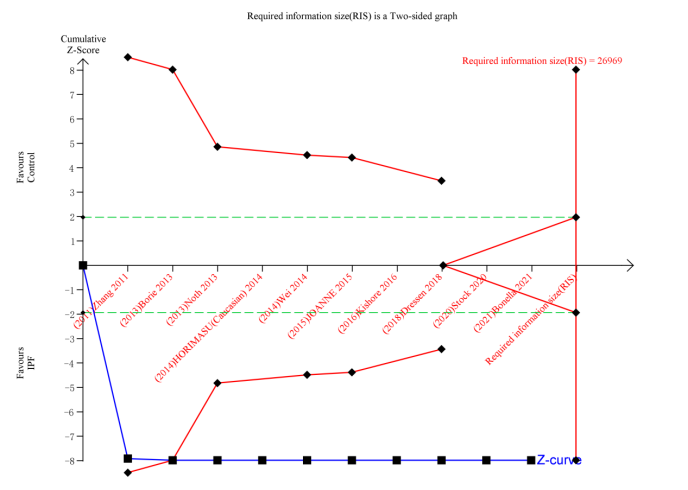
**

Figure S8a Trial sequential analysis of MUC5B polymorphism and IPF risk in Caucasian using the dominant genetic model(GT+TT vs. GG)

**
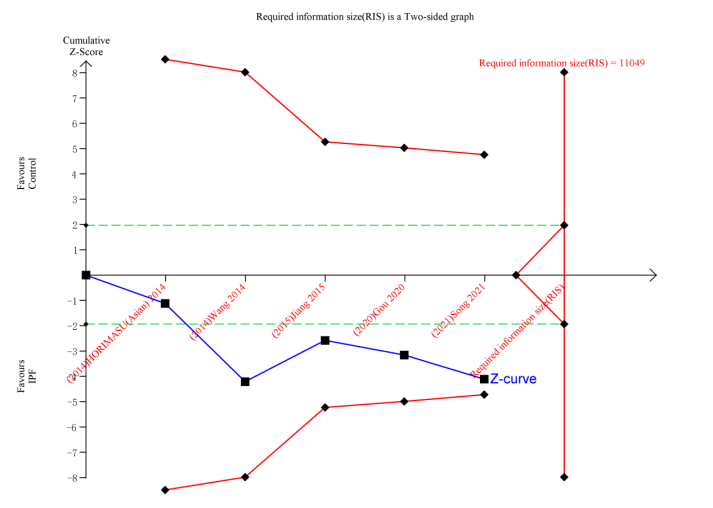
**

Figure S8b Trial sequential analysis of MUC5B polymorphism and IPF risk in Asian using the dominant genetic model(GT+TT vs. GG)

**
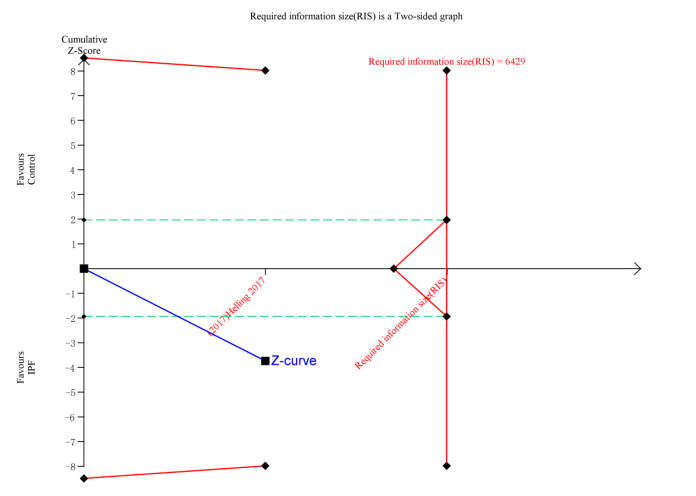
**

Figure S8c Trial sequential analysis of MUC5B polymorphism and IPF risk in Mixed using the dominant genetic model(GT+TT vs. GG)

Figure S8d Inverted funnel chart of GT+TT vs. GG of Caucasian

Figure S8e Inverted funnel chart of GT+TT vs. GG of Asian

Figure S8f Inverted funnel chart of GT+TT vs. GG of Mixed

**Figure S8 MUC5B polymorphism and IPF risk in different populations using the dominant model (GT+TT vs.GG)**

Note:Figure S6a-c:The Blue line was cumulative Z curve;The Red line was the Trial sequential monitoring(TSA) boundary or Required information size(RIS);The Green line was the Conventional boundary.(Adjusted Boundaries Print)

Figure S9a Influence analysis results of TT vs. GG+GT

******

Figure S9b TT vs. GG+GT funnel chart generated by Begg's Test(Pr > |z|=0.951 (continuity corrected))

******

Figure S9c TT vs. GG+GT funnel chart of bias generation detected by Egger's test(P>|t|=0.679)

**Figure S9 Influence analysis, Begg's Test and Egger's test results of TT vs. GG+GT**

**
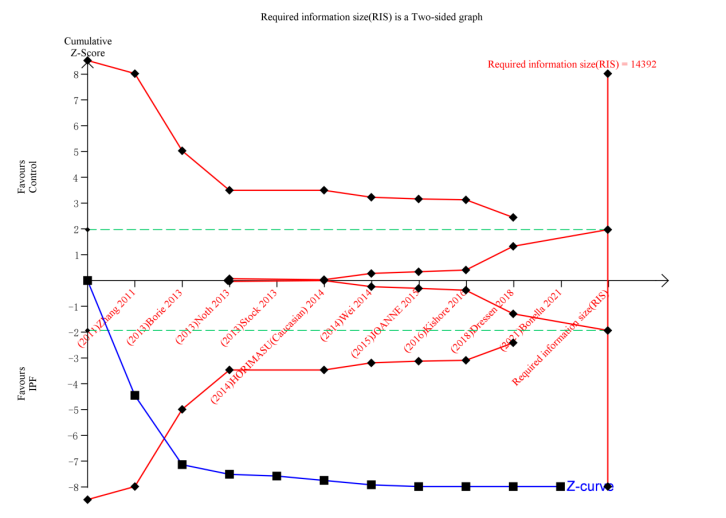
**

Figure S10a Trial sequential analysis of MUC5B polymorphism and IPF risk in Caucasian using the recessive genetic model(TT vs. GG+GT)

**
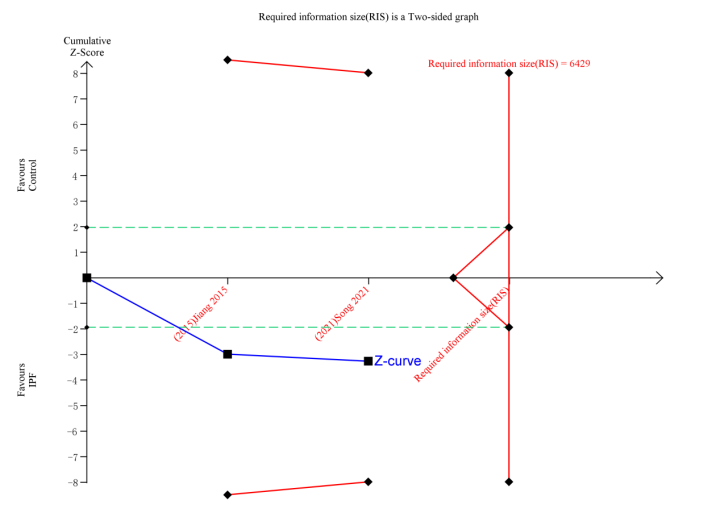
**

Figure S10b Trial sequential analysis of MUC5B polymorphism and IPF risk in Asian using the recessive genetic model(TT vs. GG+GT)

**
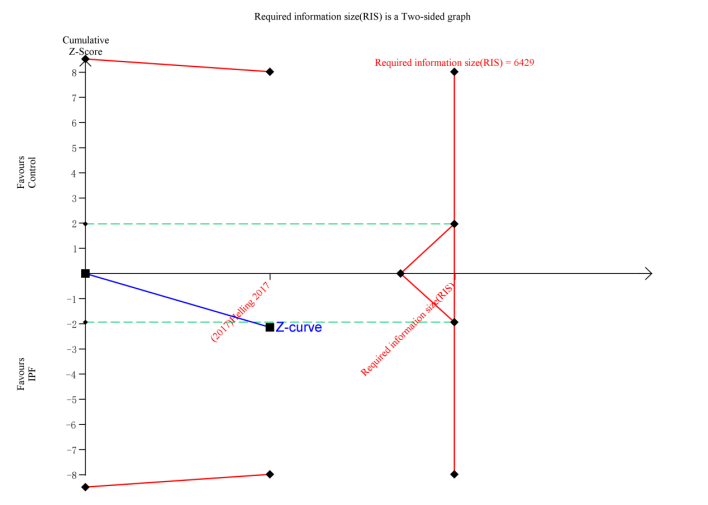
**

Figure S10c Trial sequential analysis of MUC5B polymorphism and IPF risk in Mixed using the recessive genetic model(TT vs. GG+GT)

Figure S10d Inverted funnel chart of TT vs. GG+GT of Caucasian

Figure S10e Inverted funnel chart of TT vs. GG+GT of Asian

Figure S10f Inverted funnel chart of TT vs. GG+GT of Mixed

**Figure S10 MUC5B polymorphism and IPF risk in different populations using the recessive model (TT vs. GG+GT)**

Note:Figure S6a-c:The Blue line was cumulative Z curve;The Red line was the Trial sequential monitoring(TSA) boundary or Required information size(RIS);The Green line was the Conventional boundary.(Adjusted Boundaries Print)

**Table S1 PubMed search strategy**

| **Number** | **Search Terms** |
| --- | --- |
| **#1** | Mesh descriptor: (Idiopathic pulmonary fibrosis) explode all trees |
| **#2** | ((((((Pulmonary fibrosisor[Title/Abstract]) OR Pulmonary interstitial fibrosis [Title/Abstract]) OR Interstitial lung disease [Title/Abstract]) OR IPF [Title/Abstract]) |
| **#3** | Or 1-2 |
| **#4** | Mesh descriptor: (MUC5B) explode all trees |
| **#5** | ((((((rs35705950[Title/Abstract])OR Mucin 5B[Title/Abstract] OR mucoprotein 5B[Title/Abstract]) |
| **#6** | Or 4-5 |
| **#7** | Mesh descriptor: (polymorphism) explode all trees |
| **#8** | 3 and 6 and 7 |

**Table S2 Newcastle Ottawa scale (NOS)**

| **Studies** | **Select** | | | | **Comparability^a^** | **Expose** | | | **Total score^b^** |
| --- | --- | --- | --- | --- | --- | --- | --- | --- | --- |
|  | **1** | **2** | **3** | **4** | **5** | **6** | **7** | **8** |  |
|  | **Ⅰ** | **Ⅱ** | **Ⅲ** | **Ⅳ** | **Ⅴ** | **Ⅵ** | **Ⅶ** | **Ⅷ** |  |
| Allen | ☆ | ☆ | ☆ | ☆ | ☆☆ | ☆ | ☆ |  | 8☆ |
| Bonella | ☆ | ☆ | ☆ | ☆ | ☆☆ | ☆ | ☆ |  | 8☆ |
| Borie | ☆ | ☆ | ☆ | ☆ | ☆☆ | ☆ | ☆ |  | 8☆ |
| Deng | ☆ | ☆ | ☆ | ☆ | ☆☆ | ☆ | ☆ |  | 8☆ |
| Dressen | ☆ | ☆ | ☆ | ☆ | ☆☆ | ☆ | ☆ |  | 8☆ |
| Gou | ☆ | ☆ | ☆ | ☆ | ☆ | ☆ | ☆ |  | 7☆ |
| Helling | ☆ | ☆ | ☆ | ☆ | ☆☆ | ☆ | ☆ |  | 8☆ |
| HORIMASU(Asian) | ☆ | ☆ | ☆ | ☆ | ☆☆ | ☆ | ☆ |  | 8☆ |
| HORIMASU(Caucasian) | ☆ | ☆ | ☆ | ☆ | ☆☆ | ☆ | ☆ |  | 8☆ |
| Jiang | ☆ | ☆ | ☆ | ☆ | ☆☆ | ☆ | ☆ |  | 8☆ |
| JOANNE | ☆ | ☆ | ☆ | ☆ | ☆☆ | ☆ | ☆ |  | 8☆ |
| Kishore | ☆ | ☆ | ☆ | ☆ | ☆☆ | ☆ | ☆ |  | 8☆ |
| Ley(UCSF) | ☆ | ☆ | ☆ | ☆ | ☆☆ | ☆ | ☆ |  | 8☆ |
| Ley(UTSW) | ☆ | ☆ | ☆ | ☆ | ☆☆ | ☆ | ☆ |  | 8☆ |
| Noth | ☆ | ☆ | ☆ | ☆ | ☆☆ | ☆ | ☆ |  | 8☆ |
| Peljto(Asian) | ☆ | ☆ | ☆ | ☆ | ☆☆ | ☆ | ☆ |  | 8☆ |
| Peljto(Mexican) | ☆ | ☆ | ☆ | ☆ | ☆☆ | ☆ | ☆ |  | 8☆ |
| Seibold | ☆ | ☆ | ☆ | ☆ | ☆☆ | ☆ | ☆ |  | 8☆ |
| Song | ☆ | ☆ | ☆ | ☆ | ☆ | ☆ | ☆ |  | 7☆ |
| Stock | ☆ | ☆ | ☆ | ☆ | ☆☆ | ☆ | ☆ |  | 8☆ |
| Stock | ☆ | ☆ | ☆ | ☆ | ☆☆ | ☆ | ☆ |  | 8☆ |
| Wang | ☆ | ☆ | ☆ | ☆ | ☆☆ | ☆ | ☆ |  | 8☆ |
| Wei | ☆ | ☆ | ☆ | ☆ | ☆☆ | ☆ | ☆ |  | 8☆ |
| Zhang | ☆ | ☆ | ☆ | ☆ | ☆☆ | ☆ | ☆ |  | 8☆ |

Note: A: Two stars with the highest comparability;B: Full score is 9☆.1-8:Case-control studies (CC);Ⅰ-Ⅷ:Cohort studies(CS).

1. Case definition;2: Case manifestations;3. Selection of control group;4. Definition of control group;5: Choose the most important/second most important factor;6. Determination of exposure;7. Methods for determining cases and control groups;

8: No response rate.

I: representativeness of exposure; II: selection of non exposed persons; III: Determination of exposure; IV: proof of no interesting results at the beginning; V: comparability; VI: evaluation of results; VII: long enough follow-up time; VIII: adequacy of follow-up.

**Table S3 Results of Begg's Test and Egger's Test to detect T vs.G bias in different populations**

| Ethnicity | n | Begg's | | Begg's | | cont. corr. | | Egger's | |
| --- | --- | --- | --- | --- | --- | --- | --- | --- | --- |
|  |  | score | s.d. | z | p | z | p | bias | p |
| Asian | 7 | 3 | 6.658 | 0.45 | 0.652 | 0.30 | 0.764 | 0.77 | 0.305 |
| Caucasian | 13 | 4 | 16.391 | 0.24 | 0.807 | 0.18 | 0.855 | 0.61 | 0.492 |
| Mixed | 4 | 0 | 2.944 | 0.00 | 1.000 | -0.34 | 1.000 | 1.76 | 0.197 |
| overall | 24 | 7 | 17.935 | 0.39 | 0.696 | 0.33 | 0.738 | 0.97 | 0.035 |

**Table S4 Results of Begg's Test and Egger's Test to detect TT vs.GG bias in different populations**

| Ethnicity | n | Begg's | | Begg's | | cont. corr. | | Egger's | |
| --- | --- | --- | --- | --- | --- | --- | --- | --- | --- |
|  |  | score | s.d. | z | p | z | p | bias | p |
| Asian | 2 | 1 | 1.000 | 1.00 | 0.317 | 0.00 | 1.000 | 0.53 | —— |
| Caucasian | 12 | -8 | 14.583 | -0.55 | 0.583 | 0.48 | 0.631 | 0.03 | 0.961 |
| Mixed | 1 | —— | —— | —— | —— | —— | —— | —— | —— |
| overall | 14 | -7 | 14.617 | -0.48 | 0.632 | -0.41 | 0.681 | 0.03 | 0.961 |

**Table S5 Results of Begg's Test and Egger's Test to detect GT vs.GG bias in different populations**

| Ethnicity | n | Begg's | | Begg's | | cont. corr. | | Egger's | |
| --- | --- | --- | --- | --- | --- | --- | --- | --- | --- |
|  |  | score | s.d. | z | p | z | p | bias | p |
| Asian | 5 | 4 | 4.082 | 0.98 | 0.327 | 0.73 | 0.462 | 2.73 | 0.195 |
| Caucasian | 12 | -4 | 14.583 | -0.27 | 0.784 | 0.21 | 0.837 | -0.14 | 0.858 |
| Mixed | 1 | —— | —— | —— | —— | —— | —— | —— | —— |
| overall | 17 | 0 | 15.144 | 0.00 | 1.000 | 0.00 | 1.000 | 0.35 | 0.605 |

**Table S6 Results of Begg's Test and Egger's Test to detect GT+TT vs. GG bias in different populations**

| Ethnicity | n | Begg's | | Begg's | | cont. corr. | | Egger's | |
| --- | --- | --- | --- | --- | --- | --- | --- | --- | --- |
|  |  | score | s.d. | z | p | z | p | bias | p |
| Asian | 5 | 4 | 4.082 | 0.98 | 0.327 | 0.73 | 0.462 | 1.90 | 0.212 |
| Caucasian | 10 | 3 | 11.180 | 0.27 | 0.788 | 0.18 | 0.858 | -0.23 | 0.810 |
| Mixed | 1 | —— | —— | —— | —— | —— | —— | —— | —— |
| overall | 15 | 7 | 11.902 | 0.59 | 0.556 | 0.50 | 0.614 | 0.55 | 0.449 |

**Table S7 Results of Begg's Test and Egger's Test to detect TT vs. GG+GT bias in different populations**

| Ethnicity | n | Begg's | | Begg's | | cont. corr. | | Egger's | |
| --- | --- | --- | --- | --- | --- | --- | --- | --- | --- |
|  |  | score | s.d. | z | p | z | p | bias | p |
| Asian | 2 | 1 | 1.000 | 1.00 | 0.317 | 0.00 | 1.000 | 0.45 | —— |
| Caucasian | 10 | -1 | 11.180 | -0.09 | 0.929 | 0.00 | 1.000 | 0.09 | 0.906 |
| Mixed | 1 | —— | —— | —— | —— | —— | —— | —— | —— |
| overall | 12 | 0 | 11.225 | 0.00 | 1.000 | 0.00 | 1.000 | 0.09 | 0.903 |
